# Unmasking the tissue-resident eukaryotic DNA virome in humans

**DOI:** 10.1093/nar/gkad199

**Published:** 2023-03-23

**Authors:** Lari Pyöriä, Diogo Pratas, Mari Toppinen, Klaus Hedman, Antti Sajantila, Maria F Perdomo

**Affiliations:** Department of Virology, University of Helsinki and Helsinki University Hospital, Helsinki 00290, Finland; Department of Virology, University of Helsinki and Helsinki University Hospital, Helsinki 00290, Finland; Department of Electronics, Telecommunications and Informatics, University of Aveiro, Aveiro 3810-193, Portugal; Institute of Electronics and Informatics Engineering of Aveiro, University of Aveiro, Aveiro 3810-193, Portugal; Department of Forensic Medicine, University of Helsinki, Helsinki 00290, Finland; Department of Virology, University of Helsinki and Helsinki University Hospital, Helsinki 00290, Finland; Department of Forensic Medicine, University of Helsinki, Helsinki 00290, Finland; Forensic Medicine Unit, Finnish Institute for Health and Welfare, Helsinki 00271, Finland; Department of Virology, University of Helsinki and Helsinki University Hospital, Helsinki 00290, Finland

## Abstract

Little is known on the landscape of viruses that reside within our cells, nor on the interplay with the host imperative for their persistence. Yet, a lifetime of interactions conceivably have an imprint on our physiology and immune phenotype. In this work, we revealed the genetic make-up and unique composition of the known eukaryotic human DNA virome in nine organs (colon, liver, lung, heart, brain, kidney, skin, blood, hair) of 31 Finnish individuals. By integration of quantitative (qPCR) and qualitative (hybrid-capture sequencing) analysis, we identified the DNAs of 17 species, primarily herpes-, parvo-, papilloma- and anello-viruses (>80% prevalence), typically persisting in low copies (mean 540 copies/ million cells). We assembled in total 70 viral genomes (>90% breadth coverage), distinct in each of the individuals, and identified high sequence homology across the organs. Moreover, we detected variations in virome composition in two individuals with underlying malignant conditions. Our findings reveal unprecedented prevalences of viral DNAs in human organs and provide a fundamental ground for the investigation of disease correlates. Our results from post-mortem tissues call for investigation of the crosstalk between human DNA viruses, the host, and other microbes, as it predictably has a significant impact on our health.

## INTRODUCTION

While it is accepted that humans are colonized by bacteria, the importance of which in health and disease is accumulating (1–7), it is considerably less appreciated that also a remarkable diversity of human viruses persists in our body (8–11). Indeed, the presence of many virus types infecting eukaryotic or prokaryotic cells (bacteriophages) has been unfolded via high-throughput sequencing. However, most sequencing data on the healthy human virome are derived from biological fluids (e.g. feces, respiratory secretions, urine, saliva, mucosal or dermal swabs; reviewed in Supplementary Table S1) (11–13), which provide only a fragmentary landscape of the viral populations residing within tissues. In fact, based on numerous qPCR studies from biopsies, eukaryotic DNA viruses are expected to be more prevalent in our organs (8,14–21), in contrast to bacteriophages that predominate in most secretions (22–25).

In addition to the sample type, other reasons for the overall low frequency (prevalence) of human eukaryotic DNA viruses in current metagenomic studies may be ascribed to laboratory processing: the ratios of viral lineages can be distorted by different enrichment methods (e.g. virus-like particles, multiple displacement amplification, rolling circle amplification, nuclease treatment), and the true occurrence of resident viruses may be grossly underestimated by the pooling and sequencing strategies employed (13,22,26,27).

In the present study, we integrated qPCR and hybrid-capture sequencing (targeted viromics) for quantitative and qualitative analysis of the within-host distributions of viral DNAs (vDNAs). We investigated the majority of human DNA viruses in nine organs, including the heart, kidney, liver and brain, the virome of which has been, thus far, only partially described.

Importantly, given the immune modulation necessary to sustain persistence, it is highly likely that human viruses have a significant impact on our health. These microbes not only can alter the virulence of co-infecting pathogens but also affect host gene transcription (28) and thereby contribute to the nuances of our immune phenotype (9,29,30).

This study sought to characterize the systemic distribution of the human DNA virome, including its intra- and inter-individual variations. The findings provide a comprehensive atlas of the numerous known viruses persisting within us. Importantly, we show that an understanding of the core prevalences, quantities and, most importantly, an integral analysis of different tissues are essential for discriminating virome deviations in various disease states (9,13,31–36).

## MATERIALS AND METHODS

### Study cohort

The cohort consisted of 31 recently deceased Finnish individuals with an age range of 36–85 years (mean 67). The manners of death were disease (*n* = 23), occupational disease (*n* = 1), injury (*n* = 5) or suicide (*n* = 2). Further details are presented in Supplementary Table S2. None of the individuals had a documented clinical condition etiologically related to viruses at death.

### Tissue samples

Skin (right anterior femoral), brain (right frontal lobe), colon (ascending), liver (left lobe), lung (right upper lobe), heart (right atrium), kidney (right, superior segment), whole blood (right femoral vein) and pulled hair samples (scalp) were collected at the post-mortem examination. An additional facial skin sample was collected from one individual with a history of shingles five weeks before death. All the samples were stored immediately at −20°C in 20% DMSO. Sterile disposable scalpels and forceps were used to process each tissue.

### DNA extraction

**Figure 1.**
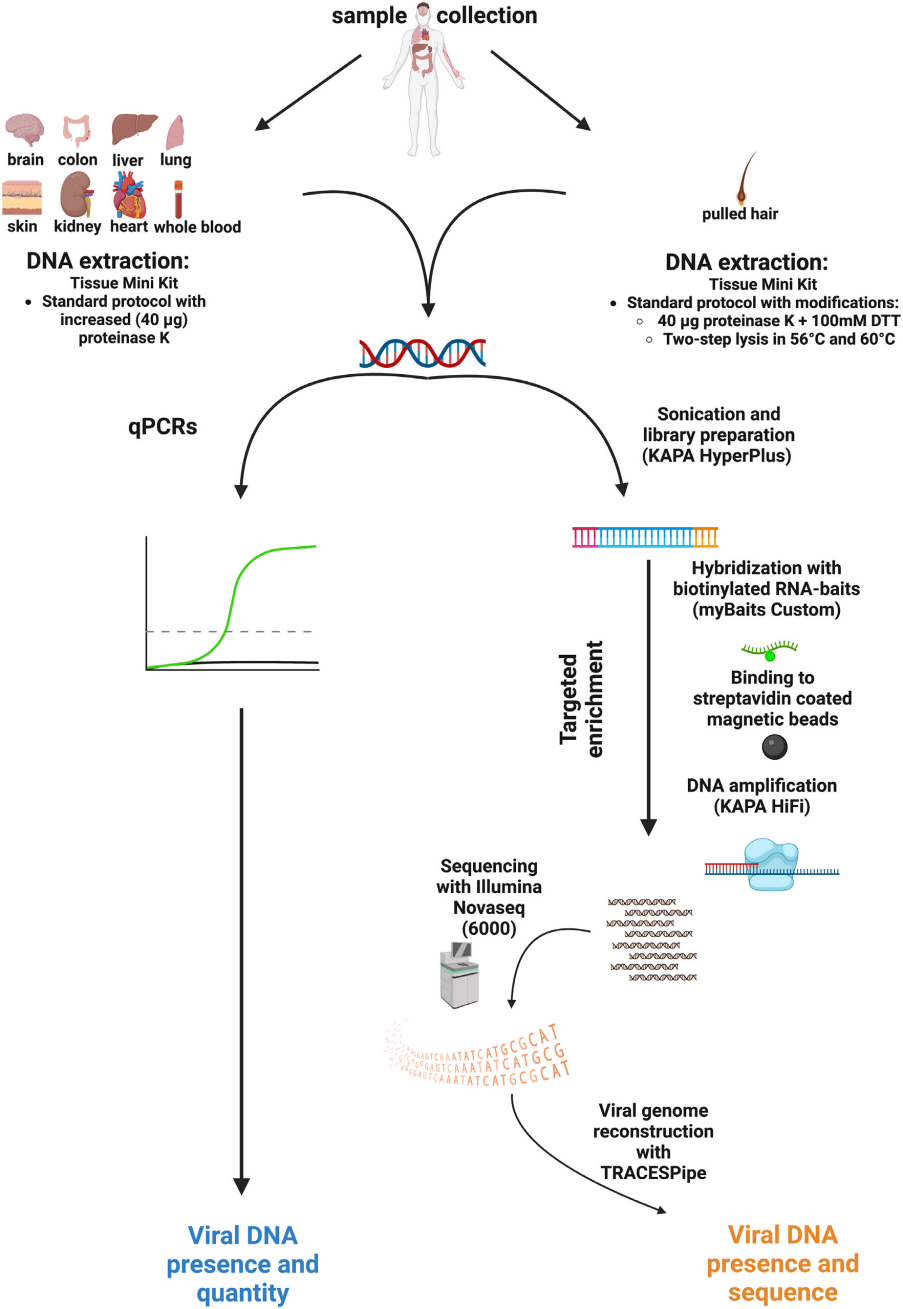
Flow diagram of the study design. Figure created with BioRender.com.

The total DNA was extracted within one year of sample collection with the DNA Mini Kit (Qiagen) following the manufacturer's instructions for tissue samples without RNAse treatment (Figure 1). For efficient lysis, the tissues were incubated overnight (o/n) at 56°C with shaking. Additional proteinase K (up to 60 μg) was used if the lysis was incomplete. The hair samples were first incubated o/n with 100 mM dl-dithiothreitol (DTT, >98% purity, Sigma Aldrich) and proteinase K at 56°C, followed by additional proteinase K plus DTT and incubation at 60°C until the lysis was complete. PBS was included as a negative control in each extraction round.

### Viral DNA quantification

The DNAs of parvovirus B19 (B19V), Torque teno viruses (TTV), and the nine human herpesviruses (herpes simplex 1 and 2, varicella zoster, Epstein-Barr, human cytomegalo, human herpes 6A, 6B and 7, Kaposi's sarcoma-associated virus), were quantified by qPCR as previously described (37–39). The hepatitis B virus (HBV) DNA was quantified with the HBV PCR Kit (Geneproof) according to the manufacturer's instructions. The DNAs of human polyomaviruses 1–13 were initially screened by an xMAP-based multiplex DNA assay (40), and the positive findings were confirmed by singleplex qPCRs (41–45). Additionally, the quantities of the human single-copy gene RNase P were determined as an internal control and for normalization of the viral copy numbers per million cells across samples (37). Plasmid dilution series (10^6^–10^1^ copies/μl) were used as positive controls and for quantification in the in-house assays. All qPCR reactions were performed in duplicates. The amplifications of B19V, HBV, polyoma-, and herpesviruses were performed with the AriaMx Real-Time PCR System (Agilent), while those of RNase P and TTV with the Stratagene Mx3005-P qPCR System (Agilent). The polyomavirus multiplex PCR detection was performed in a Bio-Plex 200 (Bio-Rad). The qPCR reagents, sample handling, DNA extractions, and plasmid handling were each done in separate hoods and rooms. Disposable filtered tips and negative controls (PCR grade water) were used in every qPCR run. The primers, probes, master mixes, thermal profiles, instruments and software used are specified in Supplementary Table S3.

### Viral enrichment and deep sequencing

The DNA extracts were mechanically fragmented using a Covaris E220 instrument with a fragment-target size of 200 bp and individually tagged with xGen Dual Index UMI Adapters (Integrated DNA Technologies) using the KAPA HyperPlus library preparation kit (Roche). The vDNAs of single samples were then captured by in-solution hybridization using 200 ng of RNA biotinylated oligonucleotides (myBaits Custom DNA-Seq, Arbor Biosciences), following the manufacturer′s recommendation for low input DNA (MyBaits v5 kit; Arbor Biosciences). Viral enrichment was performed on individual samples of 13 subjects (altogether 96 samples), using two consecutive rounds of hybridization. The xGen Universal Blockers-TS Mix (Integrated DNA Technologies) was used to block unspecific binding to the adapters during hybridization. The baits were 100 nucleotides long, designed with 2X tiling, and targeted the full-length sequences of 38 human DNA viruses (parvovirus B19; Torque teno viruses 1, 10 and 13; nine herpesviruses; polyomaviruses 1–13; hepatitis B virus; papillomavirus types 2, 6, 11, 16, 18, 21, 45; bocaviruses 1–4; cutavirus; simian virus 40; and variola viruses minor and major), as described (39,46). The individually enriched libraries were quantified with the KAPA Library Quantification Kit (Roche) using Stratagene Mx3005-P qPCR System and subsequently pooled for sequencing in NovaSeq 6000 (one lane, S4, PE151, Illumina). During library preparation and viral enrichment, the libraries were amplified 3 × 13–25 cycles. The clean-up steps were performed with KAPA Pure Beads (Roche). Negative controls (PCR-grade water) were included in the library preparation, enrichment, and sequencing.

### NGS data analysis

The sequencing reads were analyzed using a custom pipeline for multi-organ virus analysis, TRACESPipe (47). Specifically, the adapter sequences were removed using Trimmomatic (48) with a maximum mismatch that allowed a full match of 2. The palindrome and simple clip threshold were set at 30 and 10, respectively. The minimum quality score required to keep a base at the beginning and the end was fixed to 3. Low-quality data were filtered using a sliding window of 4 with mean quality of 15, and low-complexity regions were flagged with GTO (49). Reads shorter than 25 bases were discarded. The assembly was performed using iterative refinement between alignment-based and *de-novo* approaches. For the former, the reference sequence with the highest similarity was selected via FALCON-meta (50) at level 47, followed by read alignment using Bowtie 2 (51) with parameters for high sensitivity. After duplicate removal, the consensus sequences were generated with SAMtools (52) and BCFTools (53). For *de novo* assembly, metaSPAdes (54) was used. The blending was performed using five rounds between both approaches while maximizing the size and quality of the genome (More detailed description of the blending in Supplementary Text S1). The final reconstructed sequences, as well as individual sequences when in low coverage, were manually inspected and confirmed by BLAST (NCBI) (55). At a computational level, potential exogenous organisms included in the NGS samples were screened, including DNA sequences for bacteria, archaea, fungi, protozoa, and plants. Additionally, the potential inclusion of mitochondrial and plastid sequences with fewer similarities to known organisms (NCBI) was analyzed. Moreover, multiple quality controls in different phases were applied to minimize the probability of biased sequencing errors, the permanence of primers and adapters with specific similarity to the viral genomes, and the proper control and handling of low-complexity regions that otherwise would minimally affect the overall breadth and depth coverage (More detailed explanation of the quality controls in Supplementary Text S2). A virus was considered NGS-positive if at least three distinct blasted and manually confirmed viral reads (>100bp) were found in non-repetitive regions of the viral genome. Reads from viruses presenting close genetic homology (e.g. human herpesviruses 6A and B, herpes simplex viruses 1 and 2, or human polyomaviruses BK and JC) were also visually inspected with Integrative Genomics Viewer (IGV v.2.5.0).

### Hepatitis B serology

The presence of hepatitis B core antibodies (total and IgM) and surface antigen was determined with the Anti-HBc II, Anti-HBc IgM, and HbsAg Qualitative II kits on an Alinity platform (Abbott).

### Statistical analysis

The figures and the analyses were performed with R-studio (v2021.09.1 + 372), SPSS (v27), Excel, and VennPainter (56). The viral copies were log_10_-transformed for analysis, and only positive samples were included in the viral-quantity statistics. One-way ANOVA was used to compare the geometric means of the viral quantities and Shannon indices between tissues, and Ryan–Einot–Gabriel–Welsch F (REGWF) was used for post-hoc analysis of the groups. The φ coefficient was calculated to estimate the correlation between viral DNA prevalences as well as co-infections. Spearman's ρ was used to estimate the intra-host association of viral DNA quantities in different tissues. *P*-values <0.05 were considered statistically significant. The Shannon index was calculated as *H* = −∑[(*p_i_*) * log(*p_i_*)], where *p_i_* is the proportion of the quantities of a particular virus found in a sample. The Bray–Curtis dissimilarity between samples was calculated with the Vegan package (v2.5–7) and illustrated in T-distributed Stochastic Neighbor Embedding (t-SNE) with the Rtsne package(57,58) in R-studio.

### Phylogeny

The phylogenetic analysis was performed using full-length genomes available from NCBI GenBank, and the assembled consensus sequences of B19V, human herpesvirus 6B (HHV-6B), Merkel cell (MCPyV), and JC polyomaviruses (JCPyV) with >70% breadth. For MCPyV, reference genomes with large deletions were excluded. For JCPyV, 29 whole genomes published from Finland and the reference sequence NC_001699 were used. The alignments were performed with MAFFT (59). For B19V, the hairpin structures' flip and flop orientation were checked manually with IGV and Geneious Prime (v. 2022.2.2). For the phylogenies, ambiguous nucleotides were included, and repetitive regions of HHV-6B were masked. The correct substitution model was first estimated with bModelTest package, and consequently, the tree was built with BEAST 2 (60) using Bayesian inference (Markov chain Monte Carlo chain length of 50E6 in all). For HHV-6B and JCPyV, the chosen model was HKY, for B19V N93, and for MCPyV GTG (with modified substitution rates:121321). All trees were run with gamma category count set to 4 and the shape and proportion invariant values set to estimated.

### Ethical statement

The Ethics Committee of the Helsinki and Uusimaa Hospital District reviewed the collection of tissue samples from deceased for virological analysis (Statement nr HUS/974/2017).

## RESULTS

### Virome of the human body

We investigated the systemic distribution of the eukaryotic DNA virome in nine organs (skin, brain, colon, liver, lung, heart, kidney, blood and pulled hair) of 31 recently deceased individuals. To this end, we integrated quantification via PCR-based methods (a total of 25 DNA viruses) and genomic characterization via targeted viromics (a total of 38 viruses), (Figure 1).

**Figure 2.**
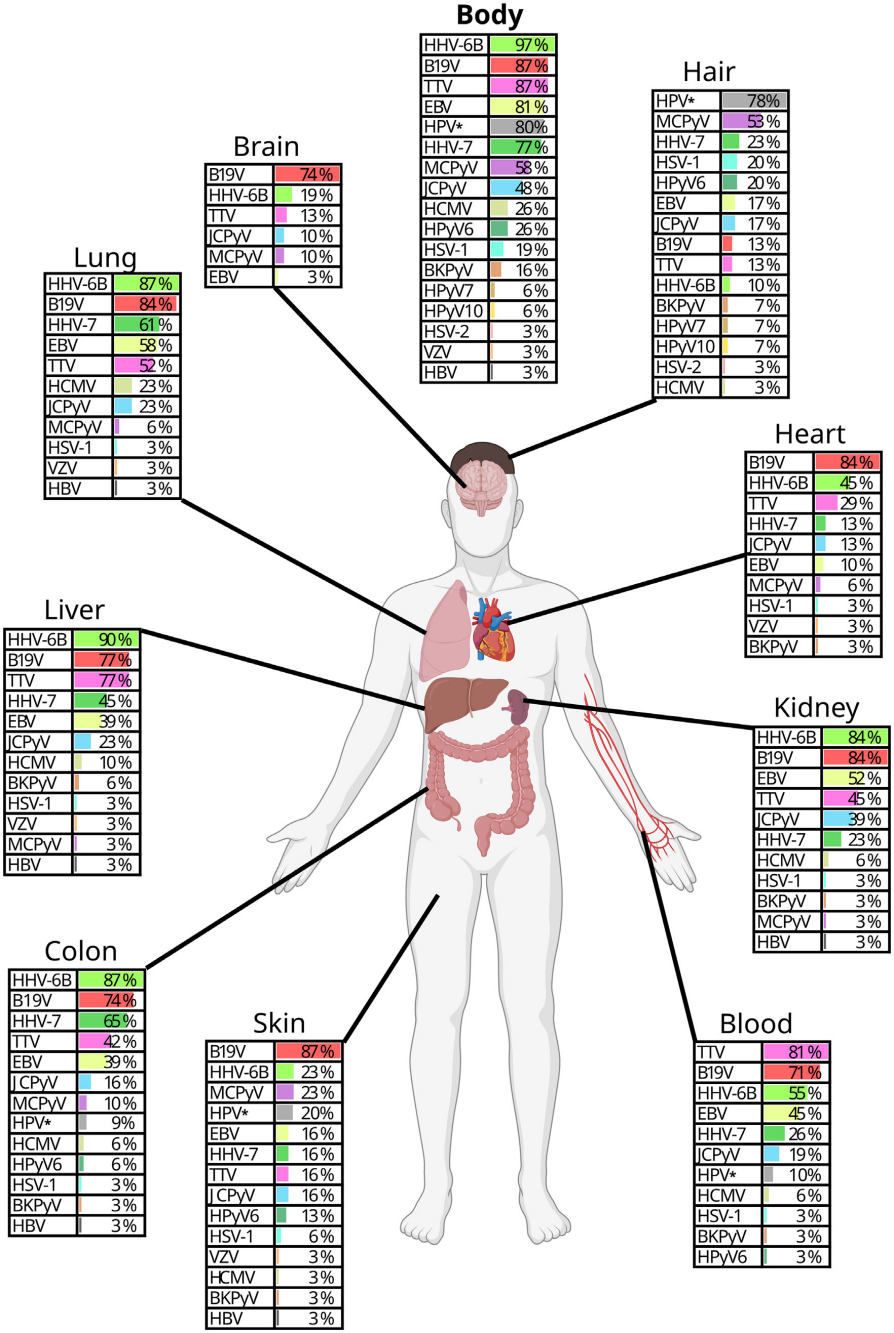
The human DNA virome. Prevalences (%) of viral DNAs in the body (≥1 tissue positive for a virus) and in different organs as determined by qPCR or NGS. *HPV prevalence was determined only via NGS from 10 individuals. Figure created with BioRender.com.

**Figure 3.**
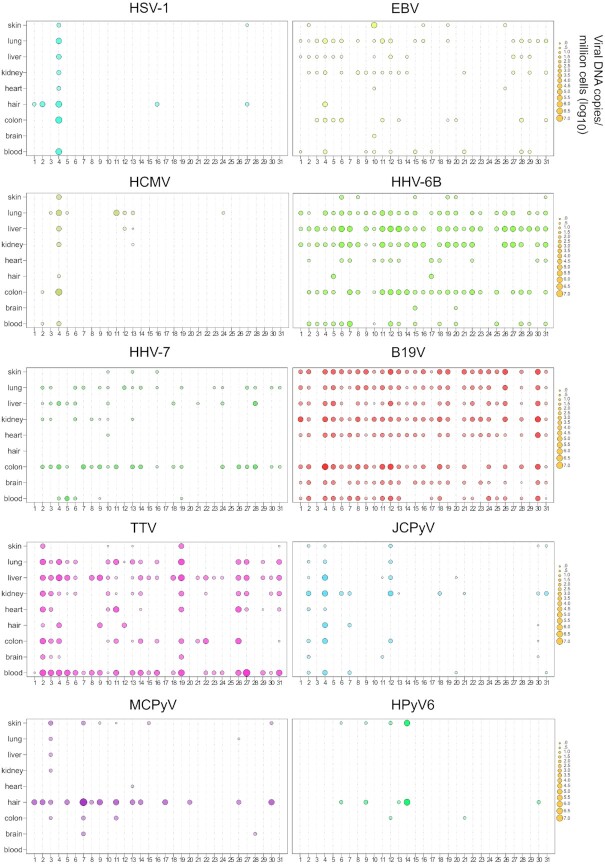
Viral distributions and quantities by qPCR. The y-axis indicates the organs and the x-axis indicates all the individuals of the cohort. Each bubble represents a positive finding, and the bubble size illustrates the viral copies/million cells (log_10_ values).

**Figure 4.**
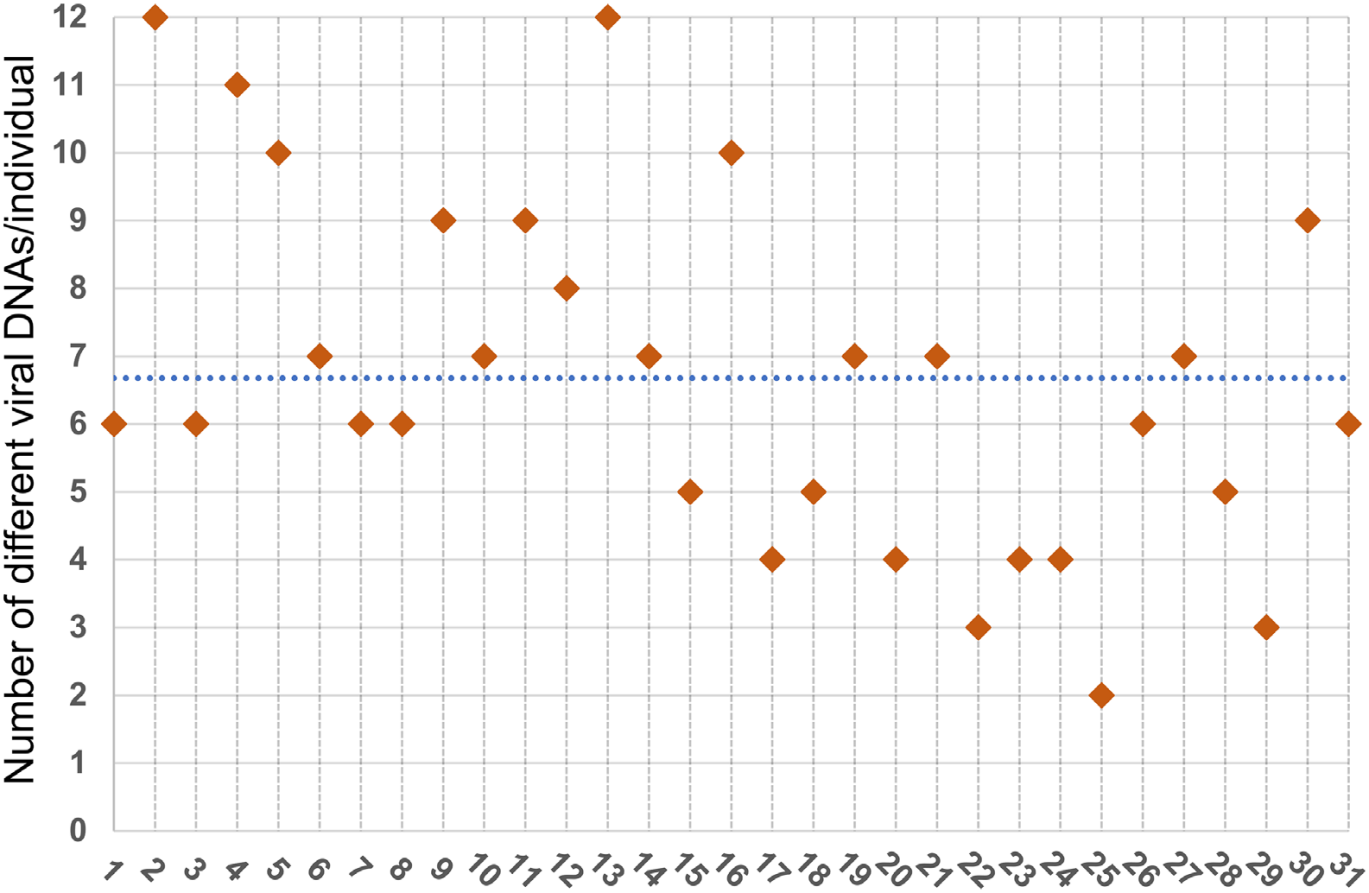
The number of viruses within an individual. The number of viruses detected with qPCR and NGS in the body (≥1 tissue positive for a virus) of the study population (mean as dashed line). On the x-axis are individuals of the cohort.

We found that the viral DNAs (vDNAs) have unique distribution profiles within the human body and across individuals (Figures 2 and 3). Out of the 279 samples analyzed, 92% were positive for vDNAs by either qPCR or NGS. We detected a total of 17 viruses (mean 6.7, range 2–12 viruses/individual), (Figure 4), of which all except human papillomaviruses (HPVs) were targeted with both methods.

The overall prevalences were high (Figure 2, Supplementary Figures S1A and S2A), with human herpesvirus 6B (HHV-6B) being the most frequent (in 97% of individuals), followed by parvovirus B19 (B19V, 87%), Torque teno viruses (TTV, 87%), Epstein-Barr virus (EBV, 85%), human herpesvirus-7 (HHV-7, 77%), Merkel cell polyomavirus (MCPyV, 58%), and JC polyomavirus (JCPyV, 48%). In addition, the prevalence of HPVs, assessed exclusively by NGS in 10 individuals, was 80%.

The copies of viral DNAs were normalized to cell counts using the human single-copy gene RNase P. The quantities are reported as viral copies per million cells (cp/mc), and the averages indicate geometric means. The viral quantities in the positive samples had a mean of 540 cp/mc. Of the most prevalent viruses, TTV and HHV-6B had the highest copies, with means of 1600 and 1100 cp/mc, respectively. The mean quantity of B19V was 550 cp/mc, while those of HHV-7, EBV, and JCPyV were in the range of 75–140 cp/mc (*P* < 0.05 One-way ANOVA), (Supplementary Figures S1A and S2B).

**Figure 5.**
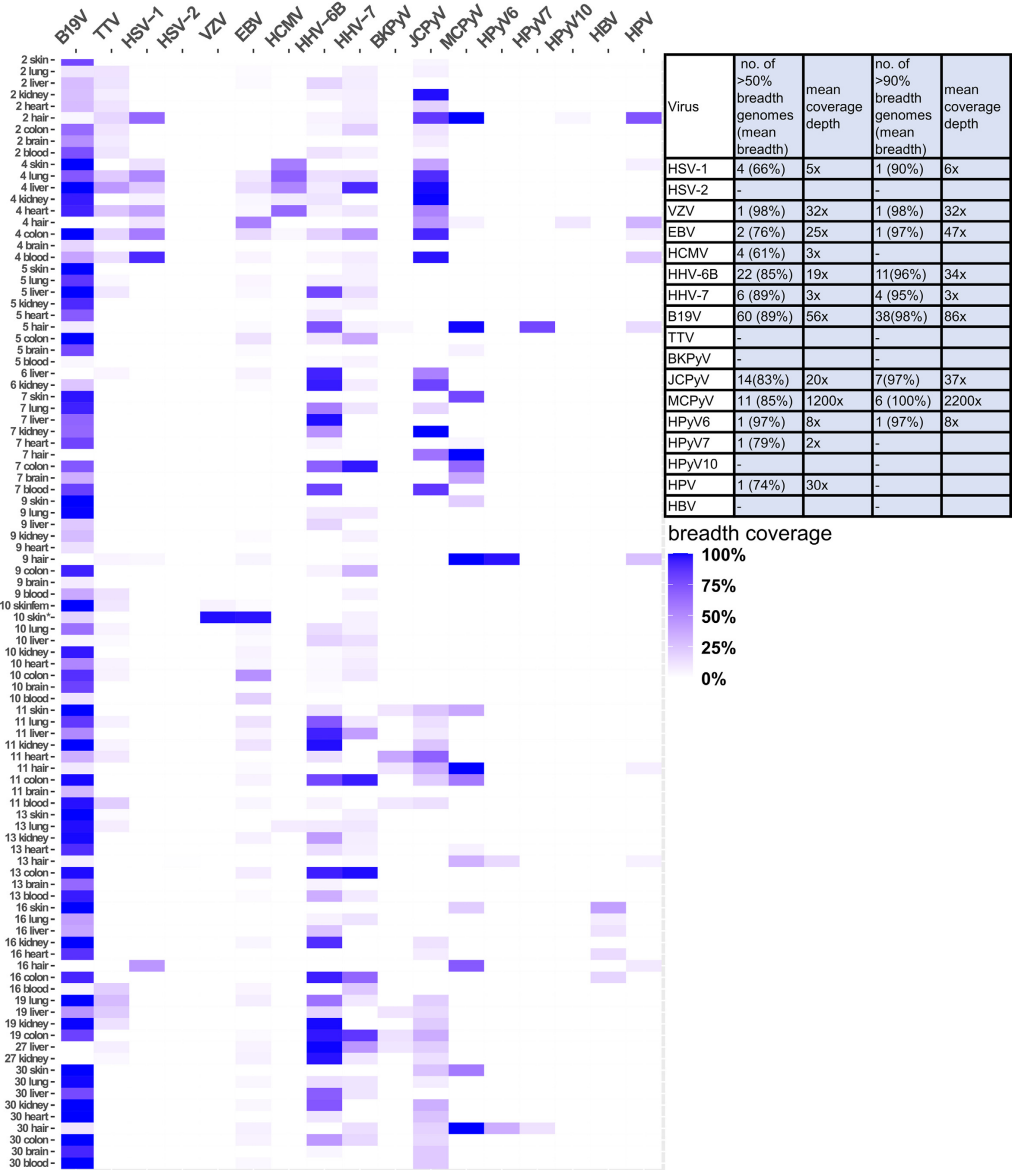
Heatmap of breadth coverages of assembled viral sequences. The y-axis indicates the samples sequenced (individual number and organ) and the x-axis indicates the respective viruses. The color intensity reflects the breadth (0–100%) of the viral genomes assembled. The table presents the number of genomes assembled with >50% and >90% breadth. *10 skin refers to the sample taken from the face.

**Figure 6.**
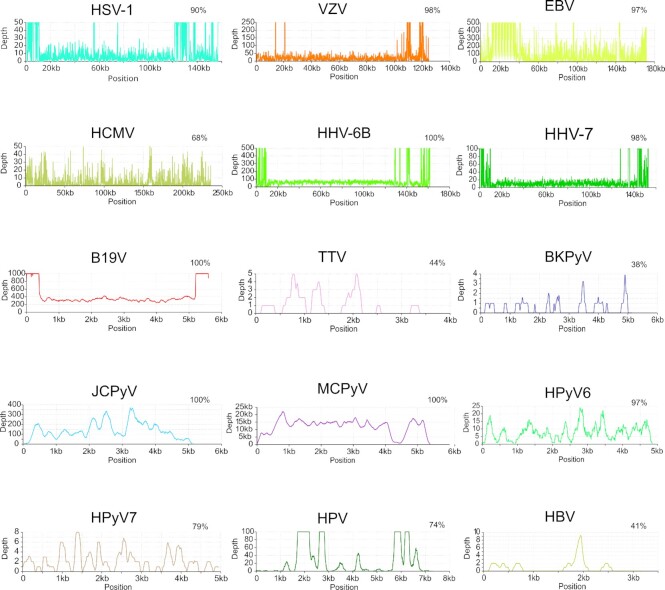
Representative coverage profiles of reconstructed viral genomes. The x-axis indicates genome position and y-axis indicates the depth of the reads (different scales). The percentage of breadth coverage of each genome is shown in the upper right corner. Correspondingly are HSV-1-blood; VZV-skin; EBV-skin; HCMV-lung; HHV-6B-liver; HHV-7-colon; B19V-skin; TTV-liver; BKPyV-heart; JCPyV-kidney; MCPyV-hair; HPyV6-hair; HPyV7-hair; HPV-hair and HBV-skin.

**Figure 7.**
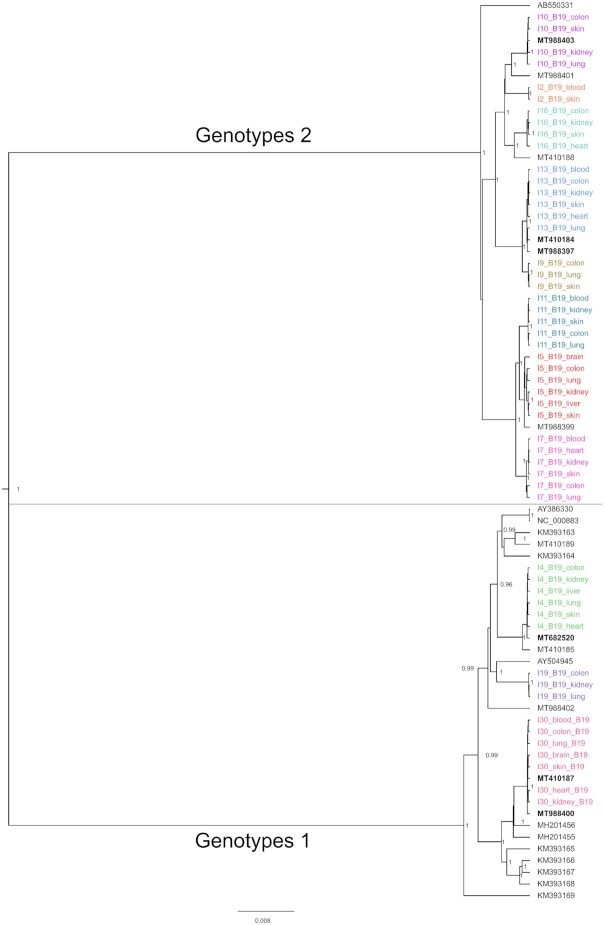
Phylogenetic tree including all the B19V genomes (>70% breadth) assembled in this study and previously published full genome sequences (including hairpins). The tree was built using Bayesian inference with Tamura-Nei (TN93) substitution model. Only posterior probabilities >0.9 are shown. Colors illustrate the sequences from each individual. Bolded sequences clustered together with sequences in this study are derived from bone (39) or bone marrow (46) of the same individuals.

We reconstructed *in silico* 129 viral genomes with over 50% breadth coverage, of which 70 were complete or near-complete (>90% breadth, Figures 5 and 6). The viral genomes assembled were unique to each individual and shared high homology across the different organs of an individual (Figure 7, Supplementary Figure S3).

**Figure 8.**
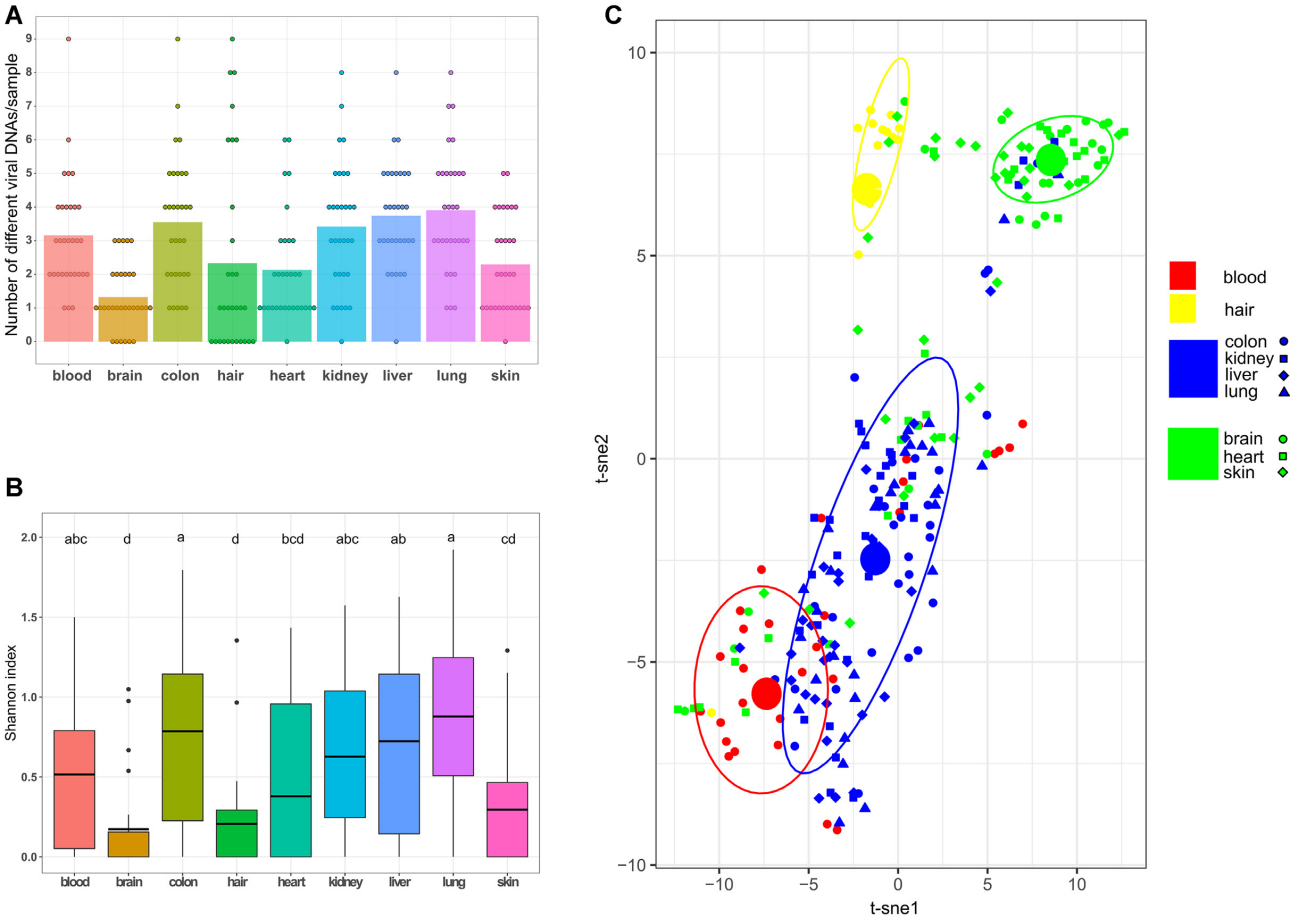
Within- and between- sample diversity. (**A**) Alpha diversity (within sample diversity) estimation by the number of viral species detected (richness) with qPCR and NGS in different sample types (mean in bar column). (**B**) Alpha diversity estimation of different organ viromes by mean Shannon index. Statistical significance was calculated by one-way ANOVA (*P*< 0.001). Post-hoc pairwise comparison of groups was done by Ryan–Einot–Gabriel–Welsh *F* (REGWF) stepwise procedure and divided into four categories (a,b,c,d) with p-value < 0.05 between categories (i.e. If two groups do not share a same letter, their mean Shannon index difference was statistically significant). (**C**) Beta diversity (between-sample diversity) was estimated using Bray-Curtis dissimilarity and plotted with t-distributed stochastic neighbor embedding (t-SNE) for visualization. Ellipses show 75% confidence interval of four observed clusters, being the solid circles the mean value of each cluster. The blue cluster consists of all colon, kidney, liver and lung samples, and the yellow of hair samples. The red cluster represents 69% of blood samples, and the green 51% of brain, heart and skin samples.

The within-sample diversities (α-diversity) were highest in the lung, liver, colon, and kidney as calculated by the richness (mean 3.9, 3.7, 3.6, and 3.4 viruses/sample, respectively) and the Shannon index (mean 0.89, 0.72, 0.79 and 0.63, respectively), (Figure 8A, B). The blood, hair, skin, and heart had a mean richness of 3.2, 2.3, 2.3 and 2.1 viruses/sample, respectively, and Shannon indices of 0.52, 0.21, 0.30 and 0.38, respectively. The brain had the lowest α-diversity, with a mean richness of 1.3 viruses/sample and a Shannon index of 0.17.

We analyzed the diversity between samples (β-diversity) by Bray-Curtis dissimilarity, which considers the presence/absence and the abundance of vDNAs in a sample. We visualized the calculated dissimilarities between samples with nonlinear dimensionality reduction t-SNE (t-distributed stochastic neighbor embedding) that models similar samples with nearby points and dissimilar samples with distant points in a two-dimensional space (Figure 8C). Based on this visualization, we identified four clusters of samples that represent low beta diversity with each other (similar vDNA composition and quantities). One cluster consisted of colon, kidney, liver and lung samples, and the second cluster of hair samples. Furthermore, 51% of brain, skin and heart samples formed a third cluster, and 69% of blood samples a fourth cluster.

We created a weighted correlation network to evaluate whether the presence (φ coefficient) and quantities (Spearman's correlation) of specific viruses in a given organ correlate with those in other organs of the same individual (Supplementary Figure S4).

The network topology was unique to each virus. The B19V DNA presence strongly correlated between organs (Supplementary Figure S4A), given that the vDNA was frequently detected in all tissues, except for hair (Figure 3). The observed correlation between the quantities of B19V suggests that the copies of viral DNA present in one organ can be predictive of the corresponding quantities in others of the same individual.

The presence of TTV and JCPyV correlated between organs, similarly to B19V (Supplementary Figure S4B, C). JCPyV, however, was detected less frequently, and the results can be strongly influenced by a few individuals with multi-tissue positivity.

The presence of EBV, HHV-6B and HHV-7 was generally restricted to specific organs, and the correlation held only between certain tissue pairs (Supplementary Figure S4D–F).

According to this analysis, in most cases, the vDNAs presence and quantities in blood did not correlate with those of other organs. Moreover, the vDNAs quantities and prevalences in blood were lower than in other organs (with the exception of the blood-borne, chronically replicating TTV), suggesting that the vDNA signal detected was, in fact, inherent to solid tissues.

### Virome of the digestive system

#### Colon

This organ harbored 13 different vDNAs (Figure 2, Supplementary Figures S1B and S2A), of which HHV-6B was the most prevalent (87%), followed by B19V (74%). Remarkably, in the colon was observed the highest prevalence of HHV-7 (65%) in any organ. TTV and EBV were detected in 42% and 39% of colonic samples, while JCPyV in 16%, and HPV, MCPyV, HCMV, HPyV6 and BKPyV in 3–11%. The mean viral copies ranged between 1200 and 1800 cp/mc for TTV, HHV-6B and B19V, and between 150–270 cp/mc for EBV and HHV-7 (Supplementary Figures S1B and S2B).

We determined the φ coefficient of all vDNA pairs from each organ to evaluate whether the co-detection of certain vDNAs was statistically significant. In the colon, this was the case for HHV-7 and B19V (*R* = 0.49; *P* = 0.005) or HHV-7 and TTV (*R* = 0.36; *P* = 0.049), (Supplementary Figure S5A).

#### Liver

There was a high prevalence of HHV-6B and B19V in the liver (90% and 77%, respectively), (Figure 2, Supplementary Figure S1C). In comparison, the prevalence of HHV-7 was lower (45%) and that of TTV higher (77%) than in the colon (Supplementary Figure S2A). HPV or HPyV6 were not detected in this organ.

The highest copies in the liver were of HHV-6B (4400 cp/mc) and TTV (2700 cp/mc), and the lowest were of EBV, HHV-7, and B19V (44–240 cp/mc) (Supplementary Figure S1C). The B19V quantities were significantly lower in the liver than in the colon (*P* < 0.05) (Supplementary Figure S2B).

JCPyV and BKPyV were often co-detected in the liver (*R* = 0.49, *P* = 0.006) (Supplementary Figure S5B).

### Virome of the respiratory system

#### Lung

We found 11 different vDNAs (Figure 2) in the lung. Here, HHV-6B and B19V had the highest prevalences (87% and 84%, respectively), followed by HHV-7 (61%), EBV (58%), and TTV (52%). The prevalences of EBV and HCMV (23%) were higher in the lung than in any other organ (Figure 2, Supplementary Figures S1D, S2A).

In the lung, TTV had the highest copies with a mean of 2700 cp/mc, while the quantities of the other viruses ranged between 32 and 630 cp/mc (Supplementary Figures S1D, S2B).

In this organ, TTV coincided with EBV (*R* = 0.49, *P* = 0.006), HHV-7 (*R* = 0.42, *P* = 0.018) or JCPyV (*R* = 0.37, *P* = 0.039), (Supplementary Figure S5C). The presence of JCPyV correlated strongly with that of HCMV (*R* = 0.48, *P* = 0.006).

### Virome of the cardiovascular system

#### Whole blood

TTV was the most frequent vDNA found in blood (81%), with also the highest quantity (mean 6200 cp/mc, *P* < 0.05) (Figure 2, Supplementary Figures S1E, S2). The TTV prevalence was followed by B19V (71%), HHV-6B (55%), EBV (45%), HHV-7 (26%) and JCPyV (19%). The mean quantities of these viruses ranged between 86–480 cp/mc. HCMV, BKPyV, HPyV6 and HPV were detected in one or two samples.

In the blood, JCPyV was co-detected with HPyV6 (*R* = 0.37, *P* = 0.040) or HCMV (*R* = 0.54, *P* = 0.002) and HHV-7 with HCMV (*R* = 0.45, *P* = 0.012), (Supplementary Figure S5D).

#### Heart

The mean viral quantities were overall low (140–850 cp/mc) in the heart. B19V was the most prevalent (84%), followed by HHV-6B (45%) (Figure 2). In contrast to blood, TTV was found in only 29% of the heart samples (Supplementary Figures S1F and S2A).

In the heart, the co-detection of JCPyV and HHV-6B (*R* = 0.42, *P* = 0.017), JCPyV and HHV-7 (*R* = 0.43, *P* = 0.017), as well as of EBV and HHV-7 (*R* = 0.53, *P* = 0.002) were statistically significant (Supplementary Figure S5E).

### Virome of the urinary system

#### Kidney

We found 11 different virus types in the kidney, of which HHV-6B (84%), B19V (84%), EBV (52%) and TTV (45%) were the most prevalent (Figure 2, Supplementary Figure S1G). Markedly, the highest prevalence (39%) and mean quantity of JCPyV (560 cp/mc) were observed in this organ (Supplementary Figure S2A). HHV-7, HCMV, BKPyV and MCPyV were detected sporadically.

In the kidney, the positivity of EBV and TTV correlated statistically (*R* = 0.52, *P* = 0.003), as did that of HCMV and JCPyV (*R* = 0.42, *P* = 0.02) (Supplementary Figure S5F).

### Virome of the central nervous system

#### Brain

This organ contained the fewest vDNAs in our cohort, with approximately 1.3 viruses/sample and a Shannon index of 0.17 (Figure 8A, B). Among the findings were B19V (74%), HHV-6B (19%), TTV (13%), JCPyV (10%), and MCPyV (10%) with a mean quantity of 160 cp/mc (Figure 2, Supplementary Figures S1H and S2).

In the brain, the co-detection of TTV and JCPyV was statistically significant (*R* = 0.43, *P* = 0.017) (Supplementary Figure S5G).

### The virome of the integumentary system

#### Skin

The skin virome was less diverse than that of the lung, colon, liver, or kidney (Figure 8A, B). The prevalence of vDNAs was, in general, markedly lower, although a total of 14 different viruses were detected (Figure 2). The highest prevalence and mean quantity was of B19V (87%, 1800 cp/mc), (*P* < 0.05) (Supplementary Figures S1I, S2). The quantities of other vDNAs in the skin ranged between 21 and 310 cp/mc.

In general, we identified polyomaviruses and HPV more often in the skin than in internal organs, with prevalences of 23% for MCPyV, 22% for HPV, 16% for JCPyV, and 13% for HPyV6. In contrast, the frequencies of herpesviruses (HHV-6B, 23%; EBV and HHV-7, 16%) and TTV (16%) were lower than in other organs (Figure 2, Supplementary Figure S2A).

In the skin, the co-detection of TTV and EBV was statistically significant (*R* = 0.52, *P* = 0.003), (Supplementary Figure S5H).

#### Pulled hair

There was a high variance in hair compared with other tissue types. While some samples had no detectable vDNAs, others had six or more (Figures 3 and 8A). We found in total 15 different viruses, of which the most frequent were HPVs (detected in 8/10 subjects by NGS), MCPyV (53%), and HSV-1 (20%); (Figure 2, Supplementary Figure S1J). The MCPyV copies in hair were remarkably high, with a mean of 24 000 cp/mc compared to 100 cp/mc in the skin (Supplementary Figures S1J and S2B).

Interestingly, we found HSV-2, HPyV7 and HPyV10 exclusively in hair. In contrast to other organs, the evidence of B19V DNA in hair (Figure 3) was limited to a few sequence reads in four samples (median 5.5 reads), (Figure 5). In one hair sample, HPV types 22 and 23 were co-detected, and in another sample HPV types 111 and 12.

### Individuals differing from the core virome

In one individual with a history of metastatic pulmonary carcinoma (Supplementary Table S2), we detected in nearly all organs the DNAs of HSV-1 (900–910000 cp/mc, mean 27 000 cp/mc), HCMV (92–620 000 cp/mc, mean 8200 cp/mc), and JCPyV (10–150 000 cp/mc, mean 3600 cp/mc), (Figure 3). These findings contrasted with those of the rest of the cohort, in which we found HSV-1 only in hair and skin, and HCMV only in lung, liver, and kidney, consistently at low prevalence. The quantities of EBV in this individual, particularly in the lung, hair, and colon, were higher than in any other subject of the cohort (11 000, 6700 and 1800 cp/mc, respectively). Furthermore, this individual was positive by NGS for HPV-122 in blood, hair, colon, and skin; for HPV-105 in blood and for HPV-111 in hair. Via NGS, we reconstructed HSV-1 genomes from blood, colon, and lung with breadth coverages of 90%, 57% and 52%, respectively, as well as HCMV genomes from the heart, liver, lung, and skin with breadth coverages of 66%, 52%, 69% and 56% (Figures 5 and 6). Moreover, from this individual, we assembled the genomes (with respective coverage breadths) of JCPyV in the kidney (100%), liver (99%), blood (97%), colon (92%), lung (90%), heart (54%), hair (45%) and skin (39%).

The sole finding of VZV DNA in the cohort corresponded to an individual with metastatic mantle cell lymphoma (stage IV), (Figure 3). This subject presented with facial herpes zoster and erythema multiforme five weeks before death (of non-natural cause) (Supplementary Table S2). Although no signs of shingles were present at the external post-mortem examination, a skin sample taken from the face revealed 630 000 cp/mc of VZV but also, remarkably, 100 000 cp/mc of EBV, the highest of this virus in any sample in the entire cohort. In comparison, in the femoral skin, heart, liver, and lung, the VZV quantities were 10–20 cp/mc, and those of EBV were 13–320 cp/mc. Of note, this was the only individual positive for EBV in the brain (320 cp/mc). From the facial skin sample, we assembled near full genomes of VZV and EBV, with respective breadth coverages of 98 and 97% (Figures 5 and 6).

Another subject in our cohort was positive for HBV DNA with quantities of 630 cp/mc in the liver. In addition, we found traces of the DNA of this virus in the subject's skin, colon, heart, lung, and kidney, at copies ranging from 10 to 50 cp/mc. The blood sample was negative by both NGS and qPCR. The presence of HBV core antibodies (HBcAb +) and the absence of both IgM (HbcAbM -) and surface antigen (HBsAg -) pointed to resolved past infection. From this individual, we assembled HBV genomes from the skin, colon, heart, liver, and lung with respective breadth coverages of 41%, 18%, 15%, 12% and 8% (Figures 5 and 6).

### Sequence analysis reveals unique viral strains in each individual

We reconstructed the following viral genomes with over 50% breadth coverage (total number of genomes; mean depth coverage): B19V (*n* = 60; 56x), HHV-6B (*n* = 22; 19x), MCPyV (*n* = 11; 1200x), JCPyV (*n* = 14; 20x), HHV-7 (*n* = 6; 3x), HSV-1 (*n* = 4; 5x), HCMV (*n* = 4; 3x), EBV (*n* = 2; 25x), VZV (*n* = 1; 32x), HPyV6 (*n* = 1; 8x), HPyV7 (*n* = 1; 2x) and HPV (*n* = 1; 30x). The viral sequences generated per organ and individual are presented in Figure 5, and representative viral genome profiles are shown in Figure 6. The B19V genomes were of genotype 2 in 73% (8/11 subjects) and of genotype 1 in 27% (3/11) of the subjects. The JCPyV genotypes were 1B in 60% (3/5) and 4 in 40% (2/5) of the subjects. Both EBV genomes were of type 1.

The viral genomes assembled were unique to each individual, excluding the possibility of a common contaminant (e.g. from reagents) (61,62). The viral consensus sequences generated from different organs of the same individual had high homology, indicating minute intra-host variation. Phylogenetic analysis from the genomes with more than 70% breadth of B19V (Figure 7), MCPyV, JCPyV and HHV-6B (Supplementary Figure S3) confirmed the relatedness between the virus sequences within an individual.

The viral genome sequences with the highest coverage from each individual were deposited in GenBank, accession numbers ON023008-ON023041.

### NGS vs. qPCR

We found an overall positive agreement of 92% (274/299; 95% Cl: 88–94%) and a negative agreement of 95% (1782/1866; 95% CI: 94–96%) between NGS and qPCR. We excluded TTV from this calculation, as its detection rate by NGS was lower (positive agreement of 41/58). This is likely due to the unsatisfactory representation of the capture baits of this highly diverse virus species.

The breadth and depth of the assembled viral sequences correlated significantly with the qPCR copy numbers (ANOVA, *P* < 0.001) (Supplementary Figure S6).

### Negative controls

Extraction controls and non-template controls were negative by qPCR. Negative controls extracted, enriched, and sequenced in parallel with the tissue samples, were negative by NGS. The sequences found from the negative controls were unspecific, low-complexity repeats that did not map to any viruses found in this cohort.

CRESS (circular rep-encoding single-stranded) viruses are common contaminants of NGS reagents (62). We blasted a representative of each virus species and all TTV sequences found in this study against reagent-associated CRESS sequences (MZ824231–MZ824237). Blasting revealed no significant similarity.

## DISCUSSION

The human body is inhabited by a remarkable diversity of viral communities, which, by modulating cell homeostasis, conceivably play significant roles in shaping our physiology and immune phenotype.

Among the different constituents of our virome, bacteriophages are most often described in metagenomic studies, while eukaryotic viruses have been commonly underrepresented (24,25). However, a chronic carrier state is established for many human DNA viruses early in life, with both detrimental (e.g. cancer and autoimmune diseases) (63–65) and beneficial effects (e.g. protection against infections) (66,67) linked to their long-term persistence.

One reason for the incomplete coverage of the eukaryotic virome is that research on healthy humans has understandably relied upon easily accessible biological fluids that, as described here, far from mirror the viral populations colonizing solid tissues. Another reason is the low quantities in which most human DNA viruses persist, only marginally approaching the current sensitivities of metagenomic approaches.

In the present study, we comprehensively examined 38 human DNA viruses in nine organs of 31 recently deceased individuals. Through the integration of quantitative analysis and targeted viromics, we revealed the persistence of these viruses in high prevalences and unique distributions throughout the human body, with a high degree of inter-individual variation.

We identified 17 different persisting viruses, on average 6.7 per individual. The lung, liver, kidney, and colon showed the greatest viral richness (range 3.6–3.9 viruses/organ), and the brain the lowest (mean 1.2 viruses). In general, the viral DNA quantities were low, with a mean of 540 copies per million cells.

The highest genoprevalences (≥80%) were of HHV-6B, B19V, TTV, EBV, HPV and HHV-7. MCPyV and JCPyV were detected in about half of the individuals, while the rest were observed infrequently (HSV-1, HCMV, HPyV6, BKPyV, HPyV7 and HPyV10) or only in single subjects (HSV-2, VZV and HBV). Through single-sample processing, we reconstructed in total 70 high-quality near-full genomes of B19V, HHV-6B, JCPyV, MCPyV, HHV-7, HSV-1, EBV, VZV and HPyV6 (>90% breadth coverage, median depth coverages: 25x).

The findings correlate with our cohort's age demographics and with the high seroprevalence of these ubiquitous viruses (e.g. >90% for EBV, HHV-6B, HHV7; >80% for B19V) (8,68). Indeed, primary infections by nearly all the viruses investigated in this study occur during childhood and are characterized by life-long persistence as supported by numerous qPCR studies (14–20). However, the knowledge on the prevalence, diversity, and, most importantly, on the systemic composition of the human eukaryotic virome in the organs of an individual has thus far remained scanty.

Our present findings are in striking contrast with those of earlier metagenomic studies in which the overall prevalence and genetic resolution of human DNA viruses have been markedly lower. Hence, our data emphasize the exploration of these viruses directly from tissues as fundamental, and targeted enrichment as decisive for high sensitivity of detection of the viral DNAs.

A common target of metagenomic studies has been the gut, the virome of which is inferred primarily via feces. In that sample type, only a few human DNA viruses (mainly TTV, adeno-associated virus, and adenoviruses) have been encountered, whereas herpes-, polyoma- and papillomaviruses have been detected infrequently (12,69–73). We, however, demonstrated that the colon is rich in these viruses, with DNA prevalences as high as 87% for HHV-6B and 74% for B19V, and remarkably 65% for HHV-7, the highest for this virus among all body sites.

The viral metagenome of the respiratory system has been studied primarily in samples of sputum or bronchoalveolar lavage. Among the eukaryotic DNA virome, Young *et al.* (74) and Abbas *et al.* (75) identified mostly TTV (72–100%) in addition to HHV-7, HPV, EBV and HCMV, albeit at low prevalences. We, on the other hand, found high viral diversity in the lung, most frequently detecting HHV-6B (87%), B19V (84%), EBV (58%) and TTV (52%). Remarkably, in this organ we detected the highest prevalences of EBV and HCMV (23%) in the cohort, suggesting that lung may be a niche of persistence for these two herpesviruses. Circumstantially, among seronegative solid organ transplant recipients, the risk for HCMV-related disease is highest in lung transplantation (76). An appealing prospect is to study whether the virome profile of an allograft (i.e. species, quantities, and sequences) could be a predictive marker of the risk of post-transplant reactivation (77).

The whole blood virome was analyzed by Moustafa et al. (78) using whole-genome sequencing datasets of 8240 individuals. The prevalences of viral DNAs reported were in general low, the highest being of HHV-7 (∼20%) and EBV (∼15%). In our whole blood analysis, TTV abounded (81%), in agreement with many qPCR studies (17) and a recent deep sequencing investigation (79). Importantly, TTV DNA was not comparably present in all organs, indicating that our findings are intrinsic to the tissue and not blood-derived. This is also supported by the fact that EBV, HHV-6B and HHV-7, known to persist in lymphatic cells, were more prevalent in the kidney, lung, liver, and colon than in whole blood.

The virome of the urinary tract in healthy individuals has been examined by metagenomics of urine samples. In contrast to Santiago-Rodriguez *et al.* (80), who reported a high diversity of low-risk HPVs in urine, we found no evidence of these viruses in the kidney. Instead, we found a high prevalence of B19V (84%), HHV-6B (84%), EBV (52%), TTV (45%) and JCPyV (39%). A possible explanation for the finding of HPV in urine may be shedding from the urinary tract epithelium or meatus.

The virome of the skin has been examined in numerous metagenomic studies, primarily via swabs. Although species similar to the ones found by us have previously been reported, few investigations (12,81–83) have detected as many different human DNA viruses in the skin. Interestingly, in our study, the virome compositions between the skin and pulled hairs differed extensively. In the skin, the most prevalent virus was B19V (87%), while in the hairs HPVs (78%) and MCPyV (53%). Remarkably, in pulled hair, the mean quantities of MCPyV were 240 times higher than in the skin (24 000 versus 1000 copies per million cells), and were the highest in the cohort. Furthermore, HSV-1, HSV-2, HPyV7 and HPyV10 occurred in hairs more often than in any other site. Differences in the microbiome between niches of the skin, likely due to distinct cellular constituents, have also been reported by Hall et al. (84).

Our study provides a comprehensive atlas of the distribution and quantities of persistent human DNA viruses across the human body. Further studies are warranted to dissect the cell hosts and mechanisms of persistence as well as to unmask the RNA virome (9). Moreover, longitudinal analyses are needed to assess the thresholds and triggers that may turn these viruses from mere commensals to contributors of disease. In this perspective, variations in virome composition in clinical conditions, as observed in cancer (36,85), should be comprehensively evaluated. Correspondingly, two individuals in our cohort with an underlying malignant condition exhibited profoundly distinct viral DNA profiles, differing not only in the systemic distribution but also in substantially higher quantities.

Indeed, the sole finding of VZV DNA in our cohort belonged to an individual with widespread mantle cell lymphoma (MCL, stage IV). The cancer was in remission until five weeks before death, when this subject presented with facial shingles and erythema multiforme, followed shortly by cancer recurrence. Although at the external post-mortem examination the dermatological condition appeared to be resolved, the facial skin sample had 680 000 copies per million cells of VZV-DNA. Strikingly, this sample also contained 100 000 copies of EBV-DNA per million cells, the highest in the cohort. While the co-existence of VZV and EBV in high quantities may reflect B cell migration and clonal expansion to the inflammatory site, an intriguing possibility is transactivation of these herpesviruses leading to subsequent malignant transformation of the MCL. In fact, case reports exist proposing a link between EBV and deterioration of MCL from an apparently stable course (86–88).

Another subject with metastatic pulmonary carcinoma had high quantities of HSV-1, EBV, HCMV and JCPyV in multiple organs, suggestive of systemic reactivation. This same individual also had in blood HPV types 105 and 122. The role of these viruses in this individual′s primary condition warrants further assessment, as independent associations of EBV, HPV, or JCPyV in specific lung cancer subsets have been proposed (89–91); and HPV-122 has been associated with high-risk oral and oropharyngeal cancers (92).

Through our multi-organ approach, we discovered in an individual hepatitis B virus DNA not only in the liver but also in the skin, colon, heart, and lung, albeit at ten-fold lower concentrations. This subject was not viremic, and the serological profile pointed to resolved infection. HBV DNA can be found in the liver even after serological resolution of chronic infection (termed occult HBV infection) (93,94) and extra-hepatic reservoirs such as peripheral mononuclear cells (95), kidneys (96), bone marrow (46) and bones (ancient and modern) (39,97) have been reported. Following organ transplantation, HBV reactivation from extra-hepatic reservoirs in the recipient or from hepatitis B core antibody-positive donors have been shown to correlate with poor graft survival (94,95,98). In this light, future studies should evaluate correlates of the *in situ* virome (quantities and genetic diversity in both the explant and allograft) with clinical progression and outcome post-transplantation (77).

Contamination is always a concern with sensitive methods such as NGS and qPCR. With this in mind, we took strict precautions through sample handling and processing, including negative controls in all phases. Our data refute the possibility of a carry-over laboratory or reagent contamination as we found diverse viral genomes and unique strains in each individual. The assessment of viral quantities and prevalences also showed each organ to have distinctive viral contents, a result improbable in contamination.

In conclusion, we expanded the characterization of the human virome from single body sites to a multi-organ analysis of several individuals. Through targeted viromics and qPCR, we identified highly prevalent and divergent species of the eukaryotic DNA virome in solid organs. We demonstrated that bodily fluids, although accessible to sampling, fail to deliver a comprehensive view of the numerous intracellular viruses colonizing our tissues. In addition, we provided a baseline on the persisting quantities and showed they are an essential criterion for the investigation of correlates to disease. We furthermore reconstructed numerous high-quality viral genomes, fundamental for the analysis of genetic diversity in various disease states (99).

Ultimately, our findings call for investigation of the crosstalk between human DNA viruses, the host, and other microbes, as it predictably has a significant impact on our health.

## DATA AVAILABILITY

The raw sequencing data have been deposited to the Sequence Read Archive (SRA; BioProject ID: PRJNA924035).

The code of TRACESPipe, used for reconstructing the viral genomes, can be found in Zenodo (DOI: 10.5281/zenodo.7646369).

The sequences of highest quality viral genomes from each individual (>90% breadth) have been submitted to GenBank with accession numbers ON023008-ON023041.

Any additional information required is available from the corresponding author upon reasonable request.

## Supplementary Material

gkad199_Supplemental_File
