# Supplementary material for: Unmasking the tissue-resident eukaryotic DNA virome in humans
Source: Nucleic Acids Res. 2023 Mar 23;51(7):3223–39. doi: 10.1093/nar/gkad199 (PMC10123123; doi:10.1093/nar/gkad199)
Supplement: gkad199_Supplemental_File [file gkad199_supplemental_file.pdf]

Supplementary Tables

Supplementary Table S1. Examples of eukaryotic viral DNA prevalences reported in virome studies

Study	Organ/ system	Sample type	Enrichment methods / pooling *	Library preparation + sequencing platforms	Cohort	Human DNA virus prevalence ca.
(1)	blood	whole blood	No enrichment / 6-sample pools	TruSeq Nano DNA HT kit + HiSeqX sequencer (Illumina)	8240 healthy blood donors (0-102yr.)	20%, HHV-7<br>15%, EBV<br>9%, TTV<br>5%, HHV-6B<br>1%, HHV-6A<br>0.6%, MCPyV<br>0.4%, HCMV<br><0.1% B19V, AdV, KSHV, HPyV
(2)	lung	BAL / OPW	VLP enrichment (filtration, concentration with 100kDa filter, nuclease treatment) +MDA	Nextera XT DNA library preparation kit + MiSeq (Illumina)	6 lung transplant recipients; 3 HIV-positives without antiretroviral therapy	100%, TTV<br>22%, HHV-7<br>11%, EBV<br>11%, HPV
(3)	lung	BAL	VLP enrichment (concentration with 100kDA filter, nuclease treatment) + MDA	Nextera XT DNA library preparation kit + HiSeq (Illumina)	Transplant patients (perioperative samples): 23 primary graft dysfunction, 23 healthy volunteers	76%, TTV<br>2%, HCMV<br>2%, circoviridae
(4)	skin	skin swab		Library preparation unspecified + HiSeq2000 (Illumina)	5 healthy individuals + one individual with previous MCC lesion	100% β-HPV<br>83%, γ-HPV<br>83%, MCPyV<br>33%,circoviridae<br>17%, HPyV6<br>17%, HPyV7<br>17%, HPyV9
(5)	skin	skin swab (multiple sites)		GAIIX platform (Illumina)	102 healthy individuals	77%, β-HPV<br>77%, γ-HPV<br>71%, other-HPV<br>41%, α-HPV<br>41%, HPyV<br>18%, HHV-6/7<br>18%, AdV<br>12%, TTV<br>6%, HCMV
	gut	stool				5%, α-HPV<br>5%, AdV<br>5%, unclassified circovirus<br>3%, HPyV<br>2%, HHV-6/7<br>2%, γ-HPV
(6)	skin	skin swab (multiple sites)		Nextera XT DNA library preparation kit + HiSeq (Illumina)	27 patients with DOCK8-deficiency (17 adults and 10 children)	100%, α-HPV<br>100%, β-HPV<br>100%, γ-HPV<br>100%, MCV<br>100%, α-PyV<br>100%, δ -PyV<br>93%, μ-HPV<br>67%, β-PyV<br>41%, HSV-1/2<br>33%, EBV<br>22%, TTV<br>19%, HCMV<br>7%, HHV-6/7<br>4%, VZV
(7)	skin	skin swab		KAPA HyperPlus Library Preparation Kit (Kapa Biosystems) + HiSeq 2500 (Illumina)	10 healthy individuals	20 %, HPV
	hair follicle	glue follicular biopsy			20 healthy individuals	10%, AAV
(8)	gut	stool	Sanger sequencing (384 clones were sequenced for each individual sample)	Big Dye terminator (v3.1) with ABI 3730xl	12 children with diarrhea from Australia or USA	17%, AAV<br>17%, AdV
(9)	gut	stool		454 Titanium FLX + sequencer (454 Life Sciences)	49 children with acute diarrhea from Western Africa	69%, TTV<br>24%, AAV<br>16%, HBoV<br>10%, AdV<br>4%, BuV<br>2%, HBV
(10)	gut	stool		GS FLX Titanium library preparation +	27 gastroenteritis patients from the Netherlands	7%, TTV<br>4%, EBV<br>4%, BuV

				GS Junior (454 Life Sciences)		
(11)	gut	stool		454 GS FLX Titanium platform (454 Life Sciences)	87 children with acute diarrhea from Australia	43%, TTV<br>11%, AdV
(12)	gut	stool	VLP enrichment (filtration, nuclease-treatment) + WTA2-kit (Sigma Aldrich) / 2-5 sample pools	Nextera DNA Library Prep XT kit + NextSeq 500 (Illumina)	221 children and adults from Cameroon	viral family (*viridae)* prevalence in pools<br>56%, *anello*<br>24%, *parvo*<br>24%, *adeno*<br>21%, *circo*<br>2%, *polyoma*<br>2%, *hepadna*
(13)	urinary tract	urine	VLP enrichment (filtration, cesium chloride density gradient, Amicon YM-100 protein columns [Millipore], nuclease treatment) + MDA	Ion Plus Fragment Library Kit +<br>Ion Torrent Personal Genome Machine (PGM; Life Technologies)	10 healthy and 10 with urinary tract infection	95%, HPV
(14)	urinary tract	urine	VLP enrichment (centrifugation, ultracentrifugation, nuclease treatment)	NEBNext DNA library prep kit for Illumina (New England BioLabs)<br>+ HiSeq 2000 (Illumina)	22 kidney transplant recipients (7 BK viremic and 15 BK non-viremic)	72%, BKPyV<br>50%, AdV<br>27%, JCPyV<br>27%, TTV<br>27%, β-HPV<br>9%, γ-HPV<br>5%, α-HPV<br>5%, HCMV
(15)	central nervous system	cerebrospinal fluid	VLP enrichment (filtering, cesium chloride density gradient, Amicon YM-100 protein columns [Millipore], nuclease treatment) + MDA	Nextera XT DNA Library Prep kit + MiniSeq (Illumina)	20 patients with various conditions (incl. but not limited to cancers, meningitis, encephalitis)	15 % unspecified HHV

*Whole genome sequencing if not otherwise stated. MDA = multiple displacement amplification BAL= bronchoalveolar lavage, OPW = oropharyngeal wash, VLP = viral-like particle, MCC = Merkel cell carcinoma, AAV= adeno-associated virus, AdV = adenovirus, HPyV = human polyomavirus, BKPyV = BK polyomavirus, JCPyV = JC polyomavirus, MCPyV = Merkel cell polyomavirus, HPV = human papillomavirus, HBV = hepatitis B virus HSV-1/2 = herpex simplex virus 1/2, VZV = varicella zoster virus, EBV = Epstein-Barr virus, HCMV = human cytomegalovirus, HHV = human herpesvirus, MCV = molluscum contagiosum virus, TTV = torque teno virus, HBoV = human bocavirus, BuV = bufavirus

Supplementary Table S2. Cohort Information.

Individual	Age	Gender	Post-mortem interval (days)	Underlying cause of death	Manner of death	Reported co(morbidities)
1	85	F	4	Alzheimer's disease	disease	pharynx neoplasia
2	79	M	9	traumatic fat embolism	injury	
3	78	M	6	malignant neoplasm of the larynx	disease	larynx & esophagus carcinoma
4	77	M	7	malignant neoplasm of the lung	occupational disease	pulmonary carcinoma metastasis in the brain, lymph nodes, liver
5	75	M	7	cerebral laceration	suicide	
6	75	M	12	aortic valve stenosis	disease	
7	74	M	5	cerebral contusion	injury	
8	74	F	7	suffocation by food	injury	
9	73	M	7	atherosclerotic heart disease	disease	
10	72	M	5	drowning	suicide	mantle cell lymphoma stage IV, facial zoster rash and erythema multiforme of upper body 5 weeks prior
11	71	M	4	atherosclerotic heart disease	disease	
12	70	M	6	atherosclerotic heart disease	disease	
13	70	M	5	chronic alcoholism	disease	
14	69	M	7	atherosclerotic heart disease	disease	
15	68	M	6	atherosclerotic heart disease	disease	
16	68	M	5	poisoning	injury	
17	67	F	6	atherosclerotic heart disease	disease	
18	67	M	7	atherosclerotic heart disease	disease	
19	66	M	5	hypertensive heart disease	disease	
20	66	M	11	traumatic subdural hemorrhage	injury	
21	65	F	6	chronic alcoholism	disease	
22	62	M	6	hemorrhagic gastric ulcer	disease	
23	60	M	7	atherosclerotic heart disease	disease	
24	60	F	30	atherosclerotic heart disease	disease	pharyngeal carcinoma operated
25	60	F	9	interstitial pulmonary disease	disease	
26	59	M	7	aortic aneurysm and dissection	disease	
27	58	M	7	aortic valve stenosis	disease	
28	54	F	28	chronic obstructive pulmonary disease	disease	
29	53	M	8	chronic alcoholism	disease	
30	53	F	8	chronic alcoholism	disease	
31	36	F	11	anorexia nervosa	disease	

Supplementary Table S3. Primers and Probes used for qPCRs

Target		Molarity (nM)	Gene	Master Mix	Thermal profile	Instrument and software used	Ref
**qPCRS**	**(Primer & Probe sequence (5' – 3')) ***<br>**Forward**<br>**Reverse(s)**<br>**Probe(s)**						
RNaseP	GAGGGAAGCTCATCAGTGGGG<br>CTTGGGAAGGTCTGAGACTAGGG<br>FAM-AGTGCGTCCTGTCACTCCACTC-BHQ1	900<br>900<br>250	RNase P	Universal master mix (Applied Biosystems)	95°C/10 min + 45 x (95°C/15s + 60°C/60s)	Stratagene Mx3005p + MxPro v4.10 (Agilent)	(16)
HSV-1	GTTGAGCTAGCCAGCGA<br>CGTTAAGGACCTTGGTGAGC<br>FAM-CGCGAACTGACGAGCTTTGTG-BHQ1	300<br>300<br>250	UL42	TaqPath ProAmp multiplex master mix (Thermo Fisher Scientific)		AriaMx Real-Time PCR System + AriaMx Software v.1.6 (Agilent)	(17)
HSV-2	CACACCACACGACAACAA<br>TAGTTCAAACACGGAAGCC<br>JOE-CGGCGATGACGGCAATAAA-BHQ1	400<br>400<br>200	UL23				
VZV	GCGCAAGGCTATTAGAGC<br>ACATGGCAGAAATCCCTG<br>TxRd-CGCATACCCGGAAGTTCTTCAGAT-BHQ2	200<br>200<br>150	ORF28				
EBV	CGGAAGCCCTCTGGACTTC<br>CCCTGTTTATCCGATGGAATG<br>FAM-TGTACACGCACGAGAAATGCGCC-BHQ1	200<br>300<br>300	BALF5				
HCMV	GTGYTCCGTGAATCGTTAC<br>AGTCKACCTCGATATCACAAGTCG<br>TxRd-ACCCTGCTGCCGCCAGT-BHQ2	400<br>500<br>300	UL54				
HHV-6A	CGGCCTCCAGAGTTGTAA<br>TGTCCCTTCAACTACTGAATC<br>FAM-AC[A]T[G]TTGC[T]A[G]AAA[G][A]CT-BHQ1 **<br>FAM-AC[A]T[G]TTGC[T]A[C]AAA[G][A]CT-BHQ1	500<br>500<br>100<br>100	U90				
HHV-6B	TTTGACAGGAGTTGCTGAG<br>GGATTCAGGAAAAAGGTTCTAA<br>JOE-AGGAAGCGTTTCGGTACACTTGGAG-BHQ1	300<br>300<br>200	U90				
HHV-7	CTCGCAGATTGCTTGTTG<br>GCATACACCAACCCTACTGTAA<br>TxRd-TTAGGCATCACGTTGGCATTG-BHQ2	400<br>400<br>300	U57				
KSHV	ATATACGGCGACACTGACTC<br>GAGCAGAAGGCACTTGAAG<br>JOE-CGGAGGAGCTAGCGTCAATCA-BHQ1	200<br>200<br>200	ORF9				
B19V	CCACTATGAAAACTGGGCAATA<br>GCTGCTTTCACTGAGTTCTTCA<br>FAM-AATGCAGATGCCCTCCACCCAG-BHQ1	400<br>400<br>150	NS1	Maxima Probe master mix (Thermo Scientific)	95°C/10 min + 45 x (95°C/15s + 60°C/15s + 62°C/30s)		(16)
TTV	GTGCCGNAGGTGAGTTTA<br>GGACTGGCCGGGCT<br>GGTCTGGCCGGGCT<br>FAM-TCAAGGGGCAATTCGGGCT-BHQ1	500<br>500<br>500<br>400	UTR		95°C/10 min + 45 x (95°C/ 15s/ + 55°C/60s)	Stratagene Mx3005p + MxPro v4.10 (Agilent)	(18)
BKPyV	CCTTACCCAATTTCCTTTTTGCT<br>ATACATAGGCTGCCCATCCAC<br>TGACCTTATAAACAGGAGAACCC	400<br>400<br>200	VP1		95°C/10 min + 45 x (95°C/20s + 60°C/60s)	AriaMx Real-Time PCR System + AriaMx Software v.1.6 (Agilent)	(19)
JCPyV	CTAAACACAGCTTGACTGAGGAATG<br>CATTTAATGAGAAGTGGGATGAAGAC<br>TAGAGTGTTGGGATCCTGTGTTTTCATCATCACT	300<br>300<br>200	LT		95°C/10 min + 45 x (95°C/15s + 60°C/60s)		(20)
MCPyV	CCACAGCCAGAGCTCTTCCT<br>TGGTGGTCTCCTCTCTGCTACTG<br>FAM-TCCTTCTCAGCGTCCCAGGCTTCA-BHQ1	900<br>900<br>250	LT		95°C/10 min + 45 x (95°C/5s + 60°C/60s)		(21)
HPyV6	TTGAGGAGCTGGACAAAGAGATT<br>TCTGGGAAGCTTTTGAATTGGT<br>FAM-AGGAAGATGCCTTGTCACAGAAAAGGAAATG-BHQ1	400<br>400<br>160	VP2		95°C/10 min + 45 x (95°C/15s + 60°C/60s)		(22)
HPyV7	GAGGAAGGAAACACTCCCCAGTA<br>TTCACTTCTTTTTGTAGCTCCTCAAG<br>FAM-ACTATACCTCAATGGATGCTTTTTGT-BHQ1	400<br>400<br>160	VP2				
HPyV10	TGAGAAGGCCCCGGTTCT<br>GAGGATGGGATGAAGATTTAAGTTG<br>FAM-CCTCATCACTGGGAGC-BHQ1	500<br>500<br>160	LT				(23)
HBV	Hepatitis B Virus PCR Kit (Geneproof)	Kit	ORFx	Kit	37°C/2 min + 95°C/10 min 45 x (95°C/5s + 60°C/40s + 72°C/20s)		

**Luminex multiplex PCR**	**Reverse primers 5' ends are biotinylated**<br>**Probes are 5' amine-C12**						
BKPyV	ACAGAGGTTATTGGAATAACTAG<br>ACTCCCTGCATTTCCAAGGG<br>CTTAACCTTCATGCAGGGTC	200 (FWD primer)<br><br>1000 (REV primer)	VP1	multiplex PCR mastermix (Qiagen)	95°C/15 min + 40 x (94°C/20s + 50°C/90s + 71°C/80s) + 71°C/10min	2720 Thermal Cycler (Applied Biosystems) + Bio-Plex 200 (Bio-Rad)	(24)
JCPyV	AATGAGGATCTAACCTGTGGAA<br>CTGCACCATTGTCATGAGTTGCTTG<br>ATGAATGTGCACTCTAATGG						
KIPyV	TTGGATGAAAATGGCATTGG<br>TAACCCTTCTTTGTCTAAAATGTAGCC<br>CTTGGAACAGCTAATAGTAGAATC						
WUPyV	TTGGATGAAAATGGCATTGG<br>TAACCCTTCTTTGTCTAAAATGTAGCC<br>GAGTACATACAGGGCTTTCCAG						
MCPyV	TTCCATCTTTATCTAATTTTGCTT<br>AGGCCTAGTTTTAGATTACCAGAC<br>GTAATAGGCCCACCATTTGT						
HPyV6	TTGCTTCTGGATCCAATACTGC<br>GGCCTCAGGAATTTCAGGCAA<br>TGGATGCTGGTTCATCTCTG						
HPyV7	AAGCAGCTACAACTGGGAACTT<br>GGCCTCAGGAATTTCAGGCAA<br>GCCTACCTTATCCTATGAGTG						
TSPyV	AGAATGTATGATGACAAAGGTAT<br>TCTGTAGTTTCCAGTTAGAAAC<br>TGAGGGAATGAATTTCCATATGTT						
HPyV9	ATCTATGGCTCATCCTCAGG<br>GTAGAGCTAGCAACTAGGCCT<br>AGTGCAGGGTACCACTCTC						
HPyV10	GTCCAGTTCCTACTAAAGTTCCT<br>TACATCATTGCCCATCCTTGGTT<br>GCCGGACACCACAATGACA						
HPyV11	TGAATATGATCCGTGCCAAA<br>ACTGCATCAGGGCCTACTTG<br>CCTCCTCCAACATGTGTTCC						
HPyV12	GTAATGGCACCCAAGAGGAA<br>GGGGATTTAGAAAGGCCTCA<br>CCCAGCAGTGTCCCTAAATT						
HPyV13	TGTGTGCCAAAGAAGTGTCCT<br>TCTGTCACCTGTTGGAGCATT<br>CTGATGCTACTACTGAAATTGAA						

*All the primers and probes were ordered from Sigma-Aldrich/Merck. **Locked nucleic acids are inside brackets. All qPCRs were done in a reaction volume of 25 µl, except for RNase P and human herpesvirus qPCRs which were done in 20µl. Luminex multiplex PCR was done in 20 µl reaction.

Supplementary Figures

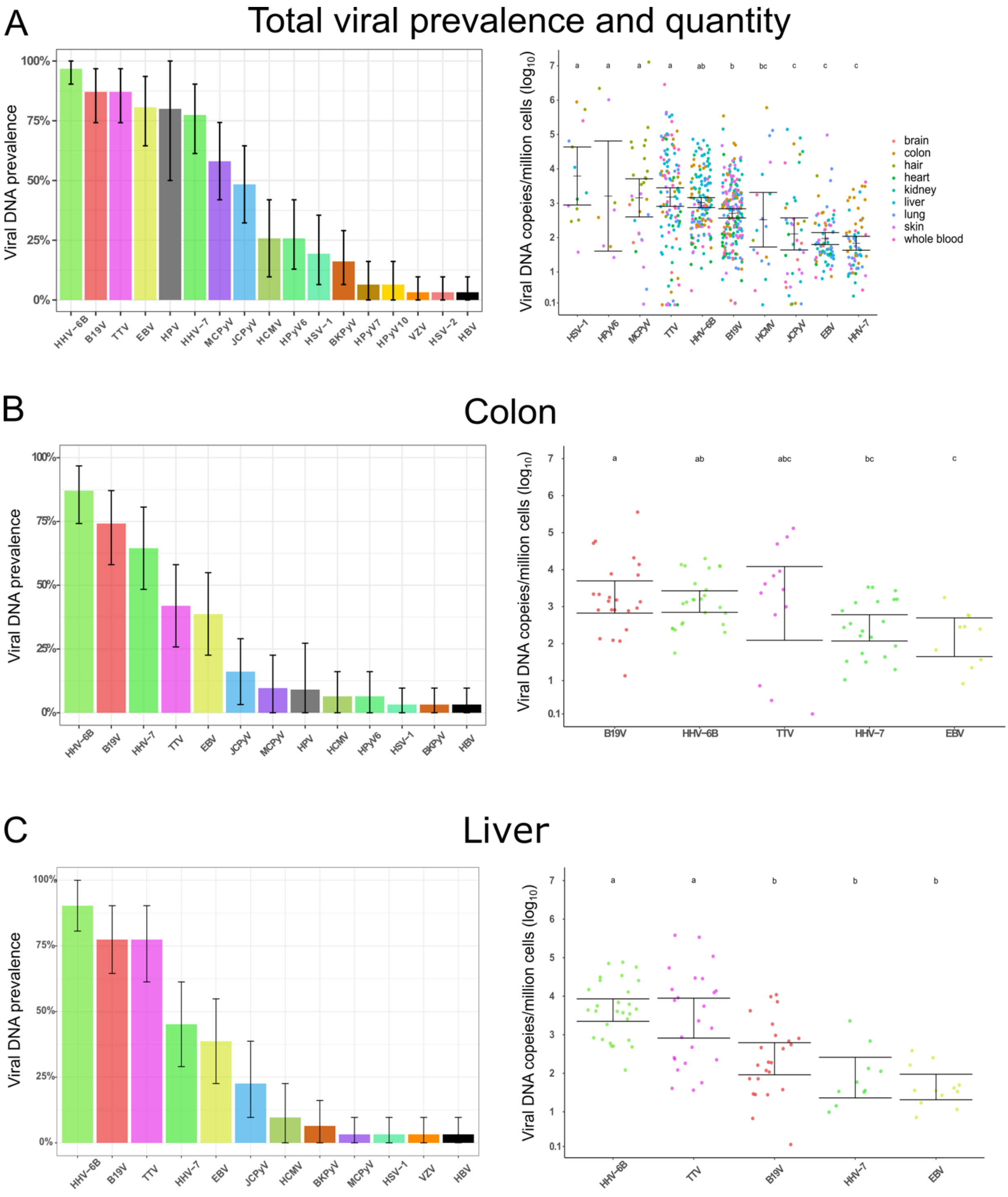

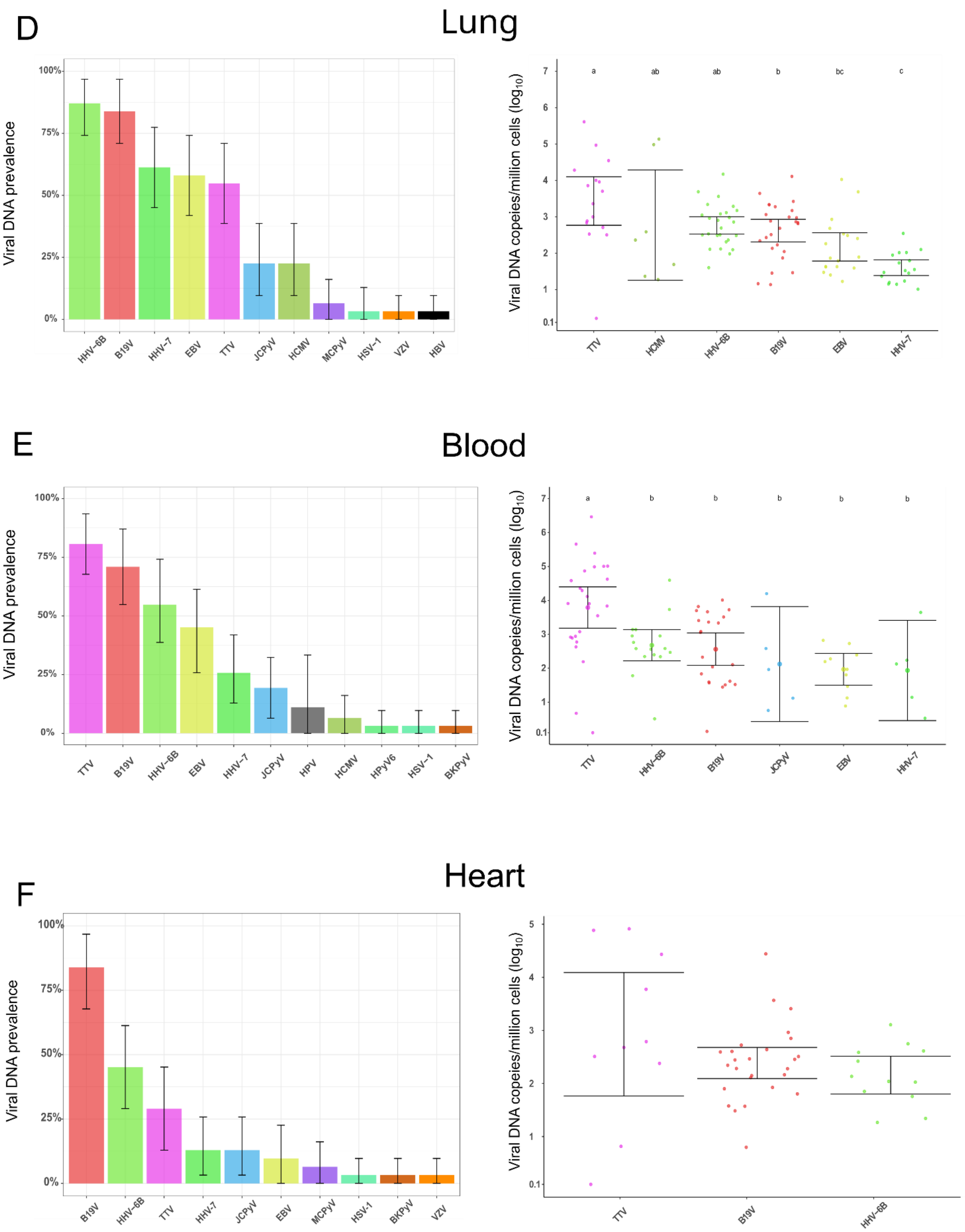

D
Lung
Viral DNA prevalence
100%
75%
50%
25%
0%
HHV-6B
B19V
HHV-7
EBV
TTV
JCPyV
HCMV
MCPyV
HSV-1
VZV
HBV
Viral DNA copeies/million cells (log10)
a
ab
ab
b
bc
c
TTV
HCMV
HHV-6B
B19V
EBV
HHV-7
E
Blood
Viral DNA prevalence
TTV
B19V
HHV-6B
EBV
HHV-7
JCPyV
HPV
HCMV
HPyV6
HSV-1
BKPyV
Viral DNA copeies/million cells (log10)
a
b
b
b
b
b
TTV
HHV-6B
B19V
JCPyV
EBV
HHV-7
F
Heart
Viral DNA prevalence
B19V
HHV-6B
TTV
HHV-7
JCPyV
EBV
MCPyV
HSV-1
BKPyV
VZV
Viral DNA copeies/million cells (log10)
TTV
B19V
HHV-6B

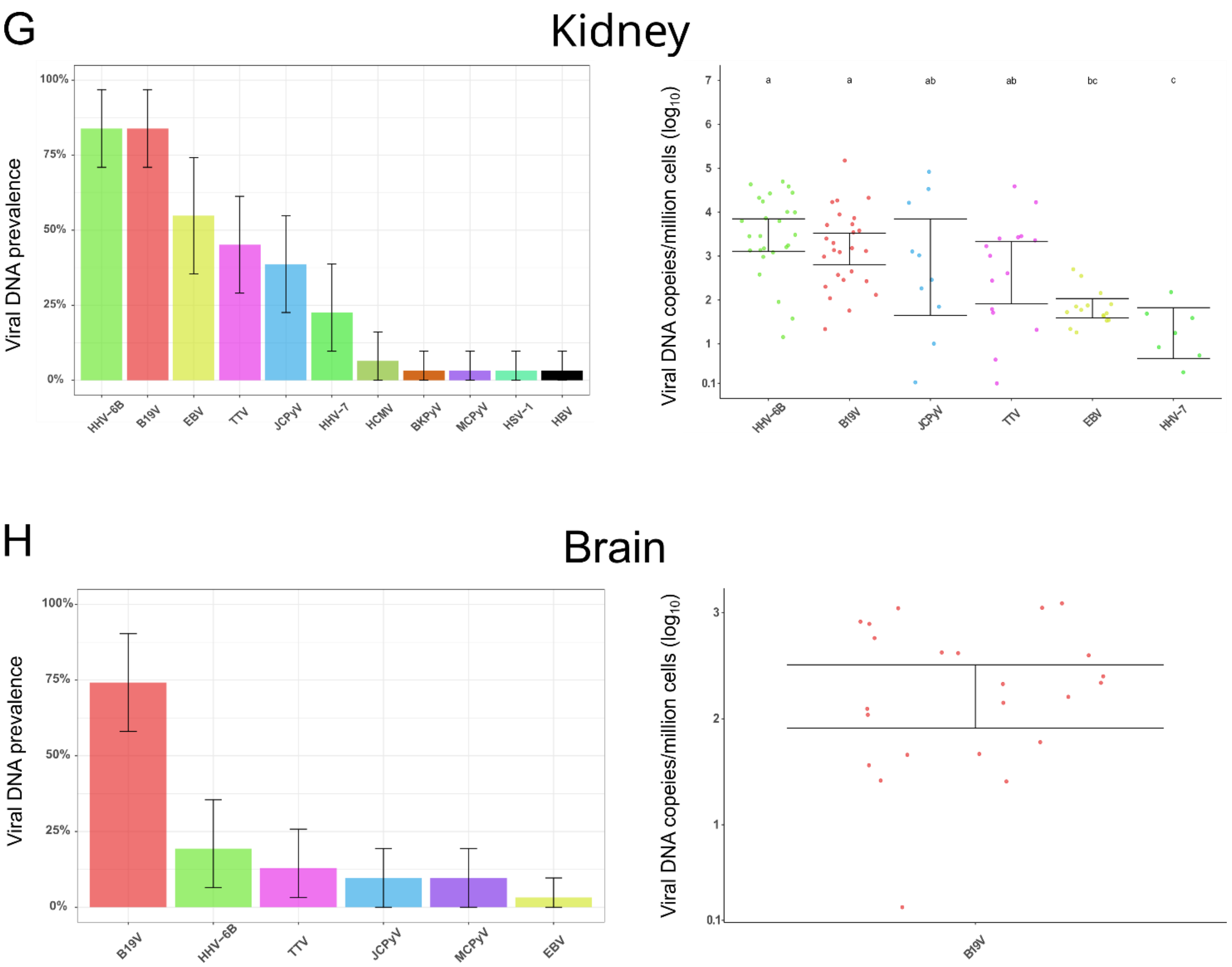
G
Kidney
Viral DNA prevalence
100%
75%
50%
25%
0%
HHV-6B
B19V
EBV
TTV
JCPyV
HHV-7
HCMV
BKPyV
MCPyV
HSV-1
HBV
Viral DNA copeies/million cells (log10)
a
a
ab
ab
bc
c
HHV-6B
B19V
JCPyV
TTV
EBV
HHV-7
H
Brain
Viral DNA prevalence
B19V
HHV-6B
TTV
JCPyV
MCPyV
EBV
Viral DNA copeies/million cells (log10)
B19V

I

Skin

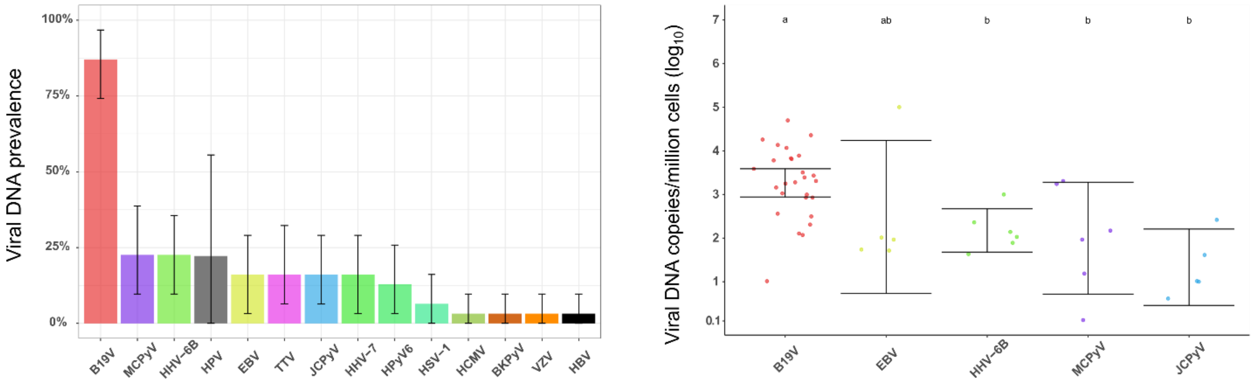

J

Hair

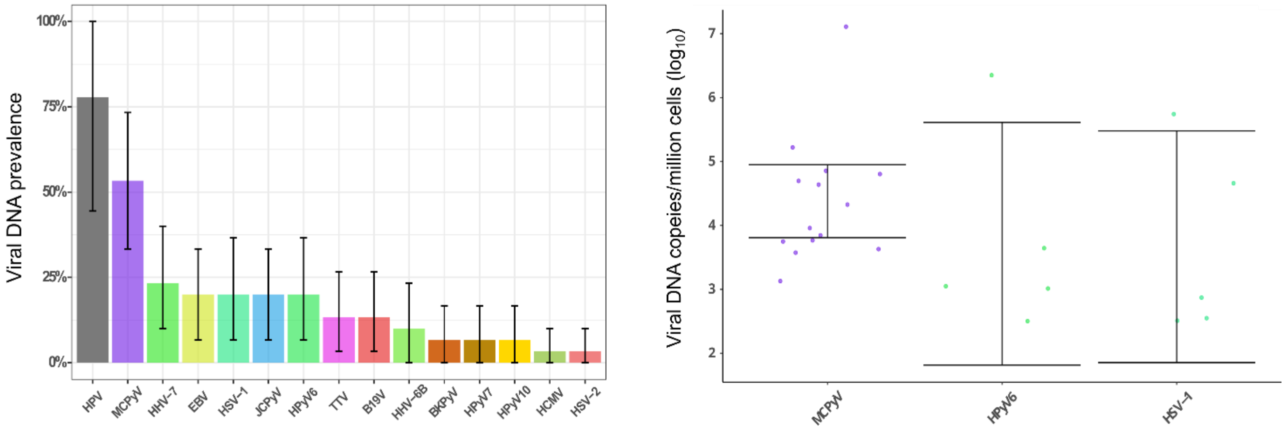

Supplementary Figure S1.Viral DNA prevalences and quantities in the body and different organs. Prevalences (left panels) in the A) body, B) colon, C) liver, D) lung, E) blood, F) heart, G) kidney, H) brain, I) skin, and J) hair as determined by qPCR and NGS, and quantities (right panels) according to qPCR. The quantity of a virus is shown if at least 5 positive samples were detected in a given organ. Error bars represent 95% confidence intervals. The geometric means of viral copies between viruses in different organs were compared with one-way ANOVA. When significant, stepwise pairwise comparison was performed with REGWF. Different categories (marked a,b,c) had statistical significance (p<0.05).

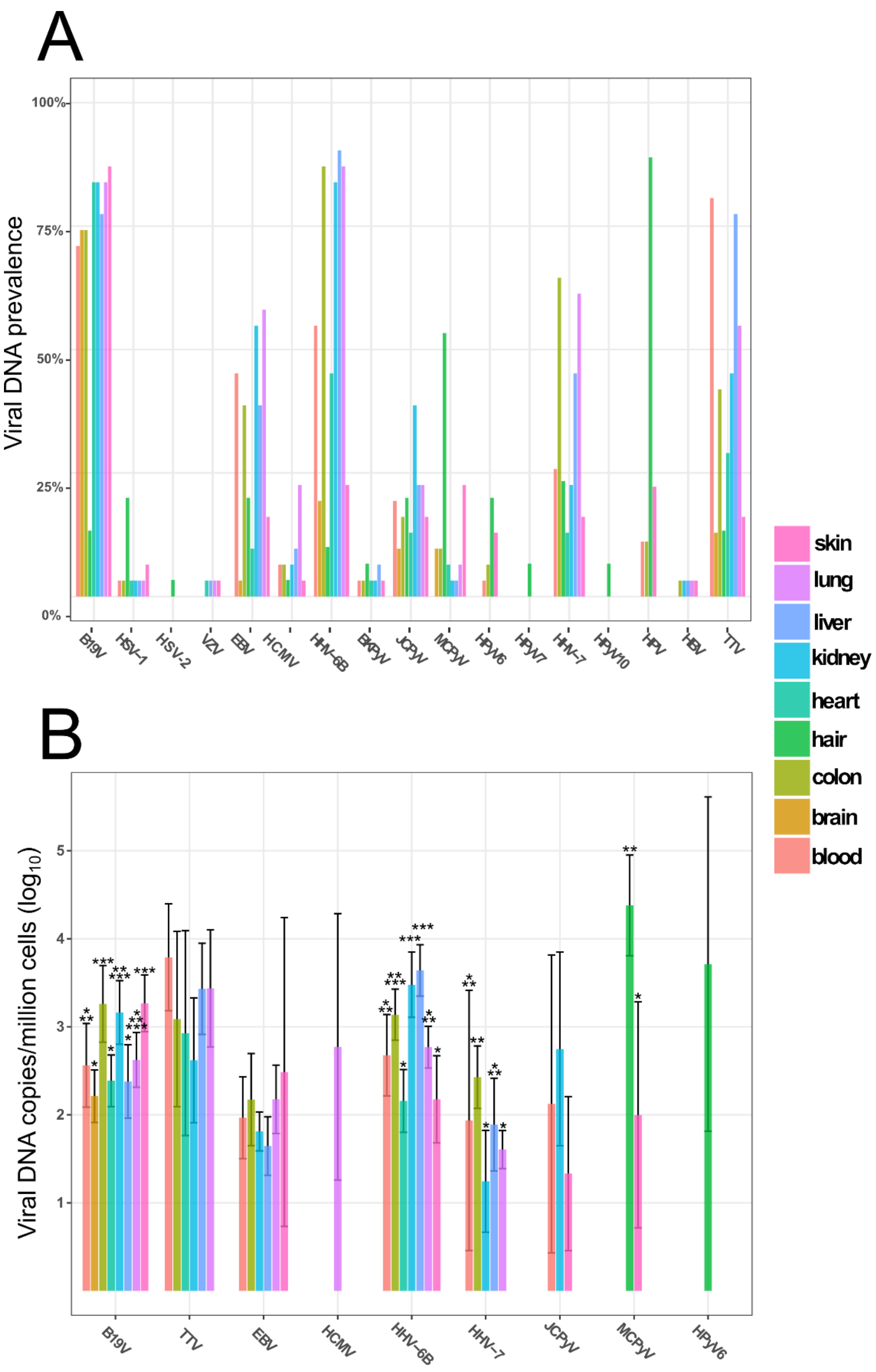

Supplementary Figure S2. Virus prevalence and quantities across organs. Side-by-side comparison of the A) prevalences and B) quantities of each viral DNA in different organs. Statistical difference of the quantities was analyzed by One-way ANOVA followed by REGW for stepwise pairwise comparisons. The different groups that showed statistical significance ($p<0.05$) are marked with asterisks.

A

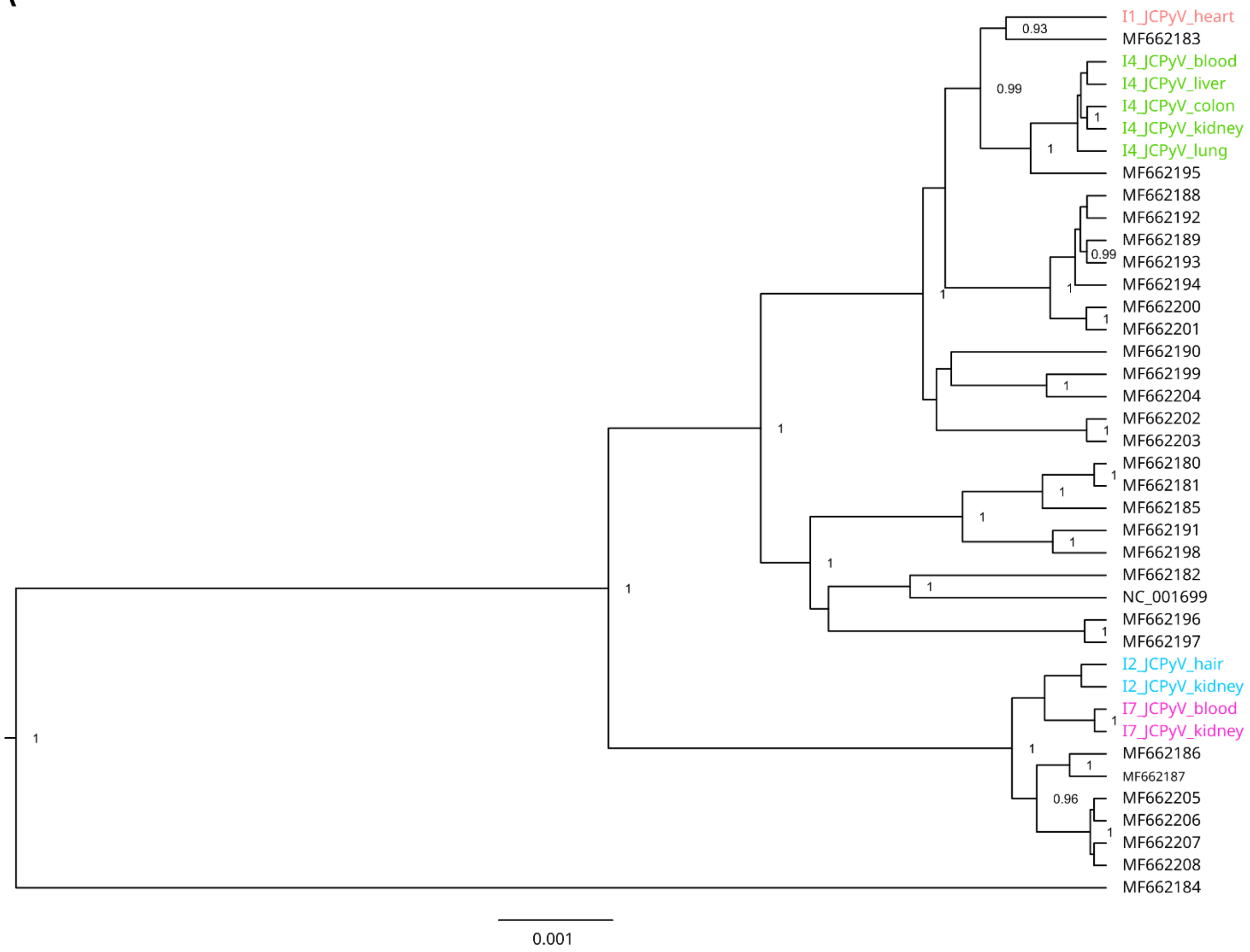
I1_JCPyV_heart
MF662183
I4_JCPyV_blood
I4_JCPyV_liver
I4_JCPyV_colon
I4_JCPyV_kidney
I4_JCPyV_lung
MF662195
MF662188
MF662192
MF662189
MF662193
MF662194
MF662200
MF662201
MF662190
MF662199
MF662204
MF662202
MF662203
MF662180
MF662181
MF662185
MF662191
MF662198
MF662182
NC_001699
MF662196
MF662197
I2_JCPyV_hair
I2_JCPyV_kidney
I7_JCPyV_blood
I7_JCPyV_kidney
MF662186
MF662187
MF662205
MF662206
MF662207
MF662208
MF662184
0.93
0.99
0.96
1
0.001

B

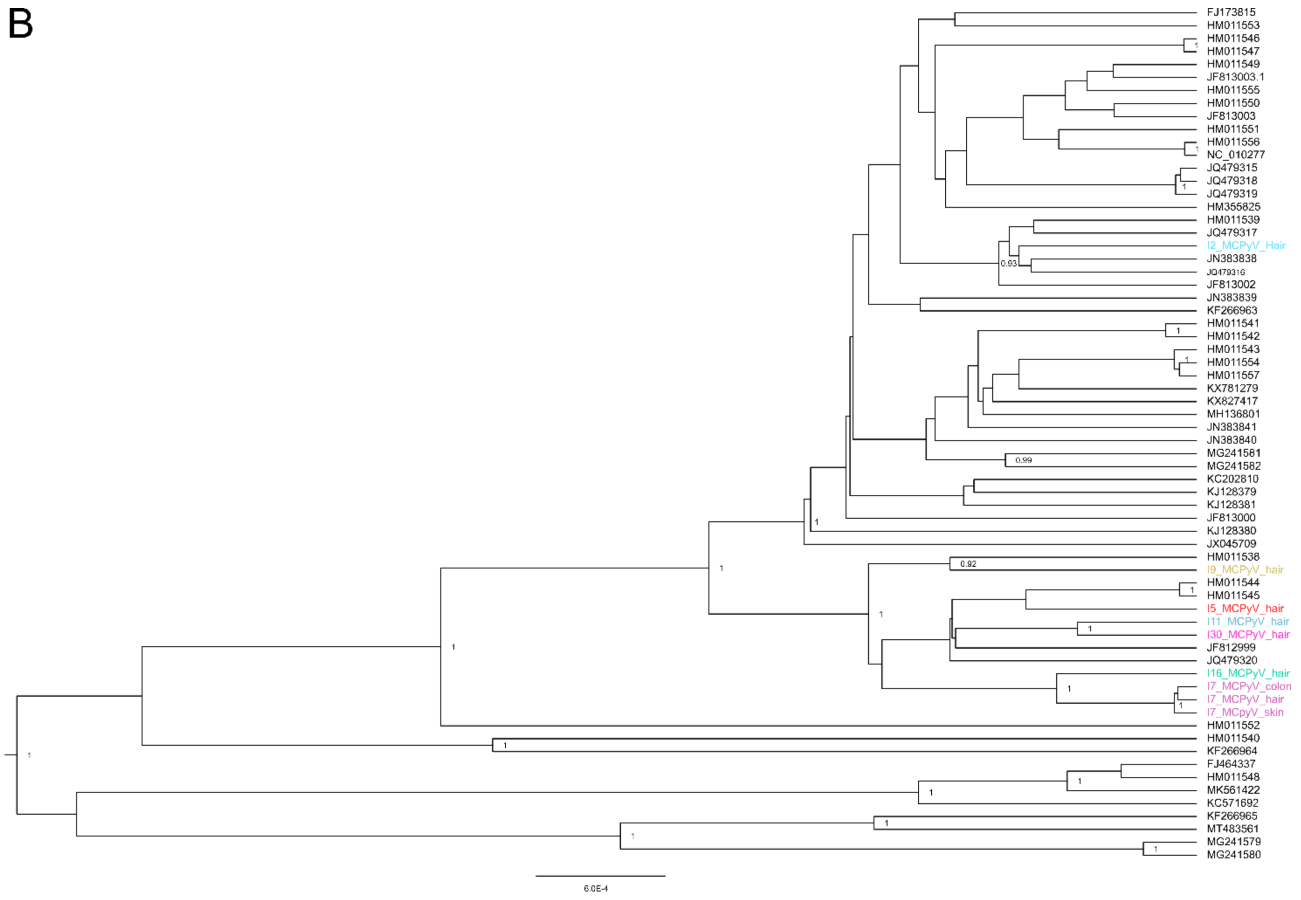
FJ173815
HM011553
HM011546
HM011547
HM011549
JF813003.1
HM011555
HM011550
JF813003
HM011551
HM011556
NC_010277
JQ479315
JQ479318
JQ479319
HM355825
HM011539
JQ479317
I2_MCPyV_Hair
JN383838
JQ479316
JF813002
JN383839
KF266963
HM011541
HM011542
HM011543
HM011554
HM011557
KX781279
KX827417
MH136801
JN383841
JN383840
MG241581
MG241582
KC202810
KJ128379
KJ128381
JF813000
KJ128380
JX045709
HM011538
I9_MCPyV_hair
HM011544
HM011545
I5_MCPyV_hair
I11_MCPyV_hair
I30_MCPyV_hair
JF812999
JQ479320
I16_MCPyV_hair
I7_MCPyV_colon
I7_MCPyV_hair
I7_MCpyV_skin
HM011552
HM011540
KF266964
FJ464337
HM011548
MK561422
KC571692
KF266965
MT483561
MG241579
MG241580
0.93
0.99
0.92
6.0E-4

C

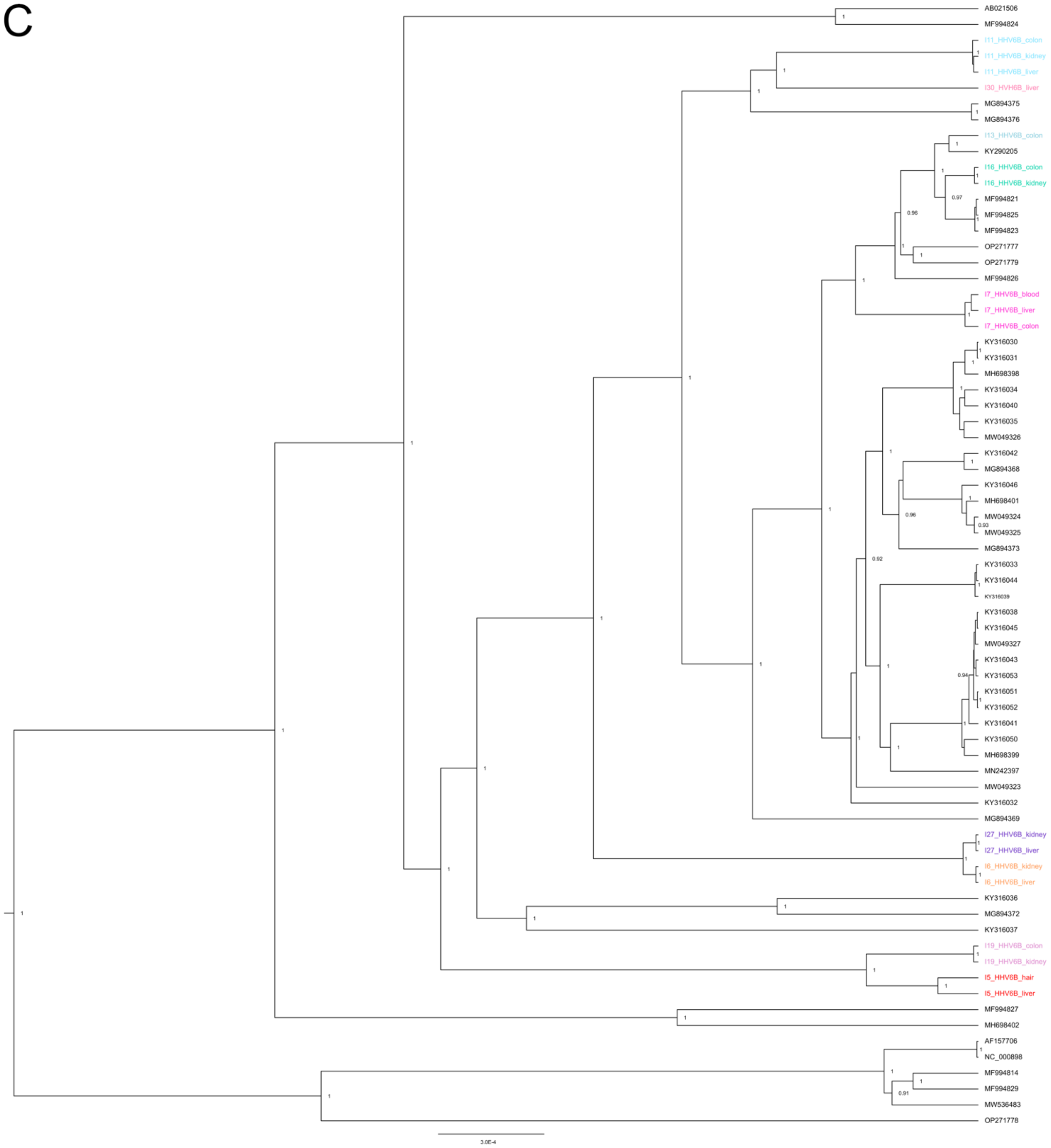

Supplementary Figure S3. Phylogenetic analysis of A) JCPyV, B) MCPyV, and C) HHV-6B. Colors represent sequences from each of the individuals of this study (>70% breadth), and in black are reference genomes from GenBank. For JCPyV, the analysis was performed using 29 full-genome reference sequences from Finland and NC_001699 using Bayesian inference with Hasegawa-Kishino-Yano (HKY) substitution model. The phylogenetic analysis of MCPyV was performed using Bayesian inference with General Time Reversible GTR (121321) substitution model and that of HHV-6B using Bayesian inference with HKY. Only posterior probabilities >0.9 are shown.

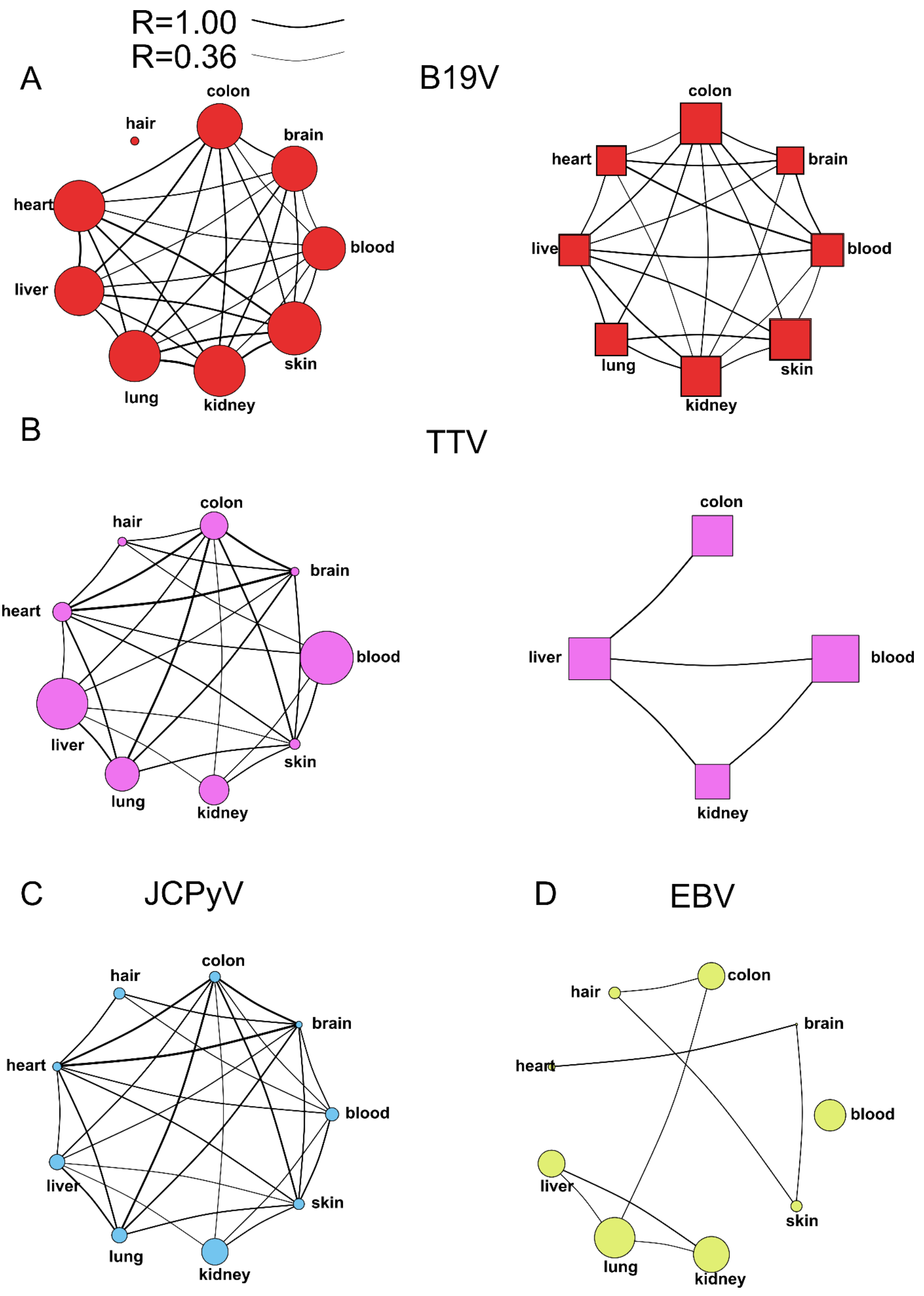
R=1.00
R=0.36
A
B19V
colon
hair
brain
heart
blood
liver
skin
lung
kidney
B
TTV
C
JCPyV
D
EBV

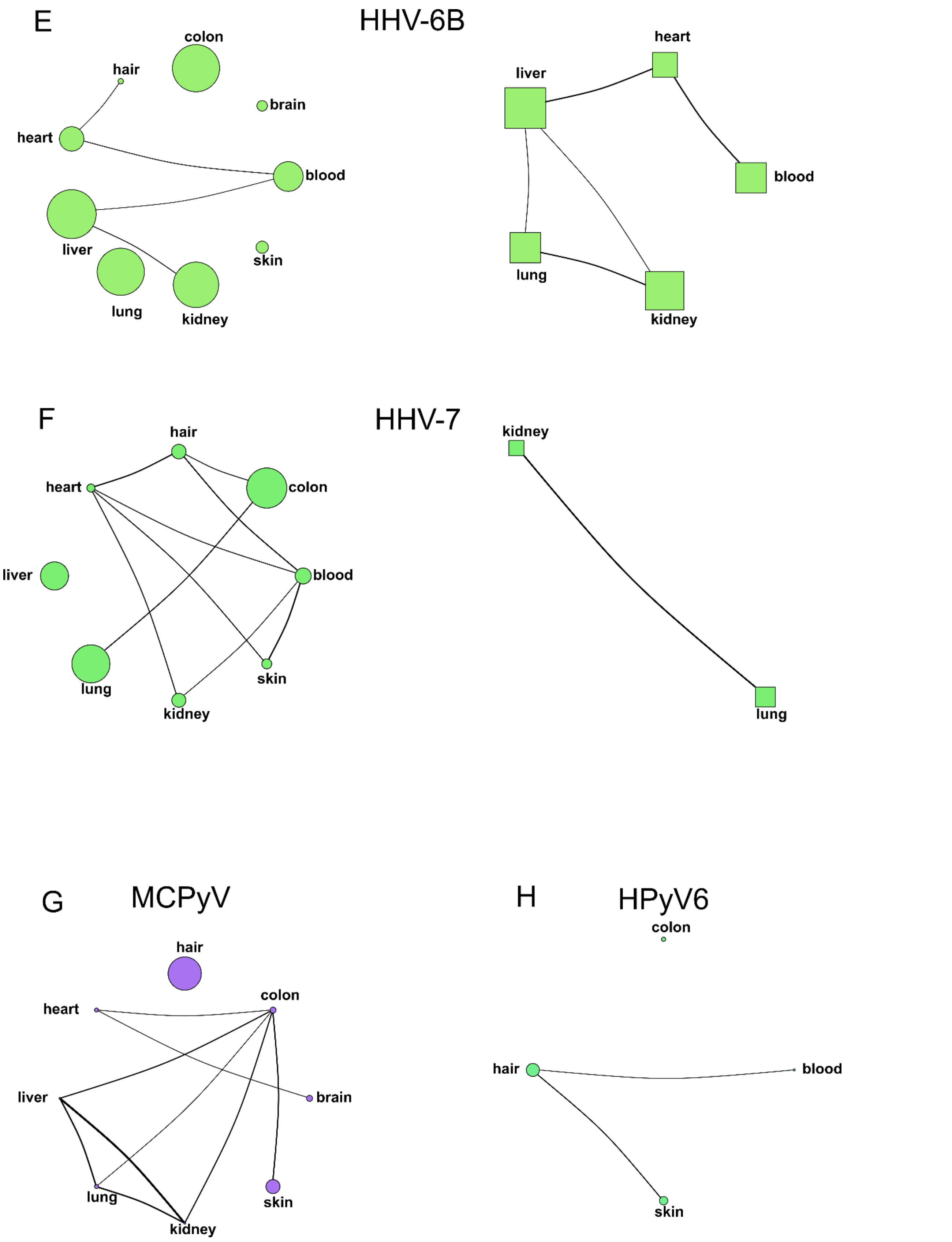

Supplementary Figure S4. Viral DNA intra-host correlation between organs. Weighted correlation network of statistically significant (p<0.05) viral prevalence (circles) or quantities (squares) of A) B19V, B) TTV, C) JCPyV, D) EBV, E) HHV-6B, F) HHV-7, G) MCPyV, and H) HPyV6 between tissues as calculated by pairwise comparisons with φ coefficient or Spearman's ρ, respectively. The width of the connecting lines (edges) represents the strength of the correlation. The size of circles and squares (nodes) indicates the percentage of viral prevalence and mean copies of viral DNA, respectively, in each organ.

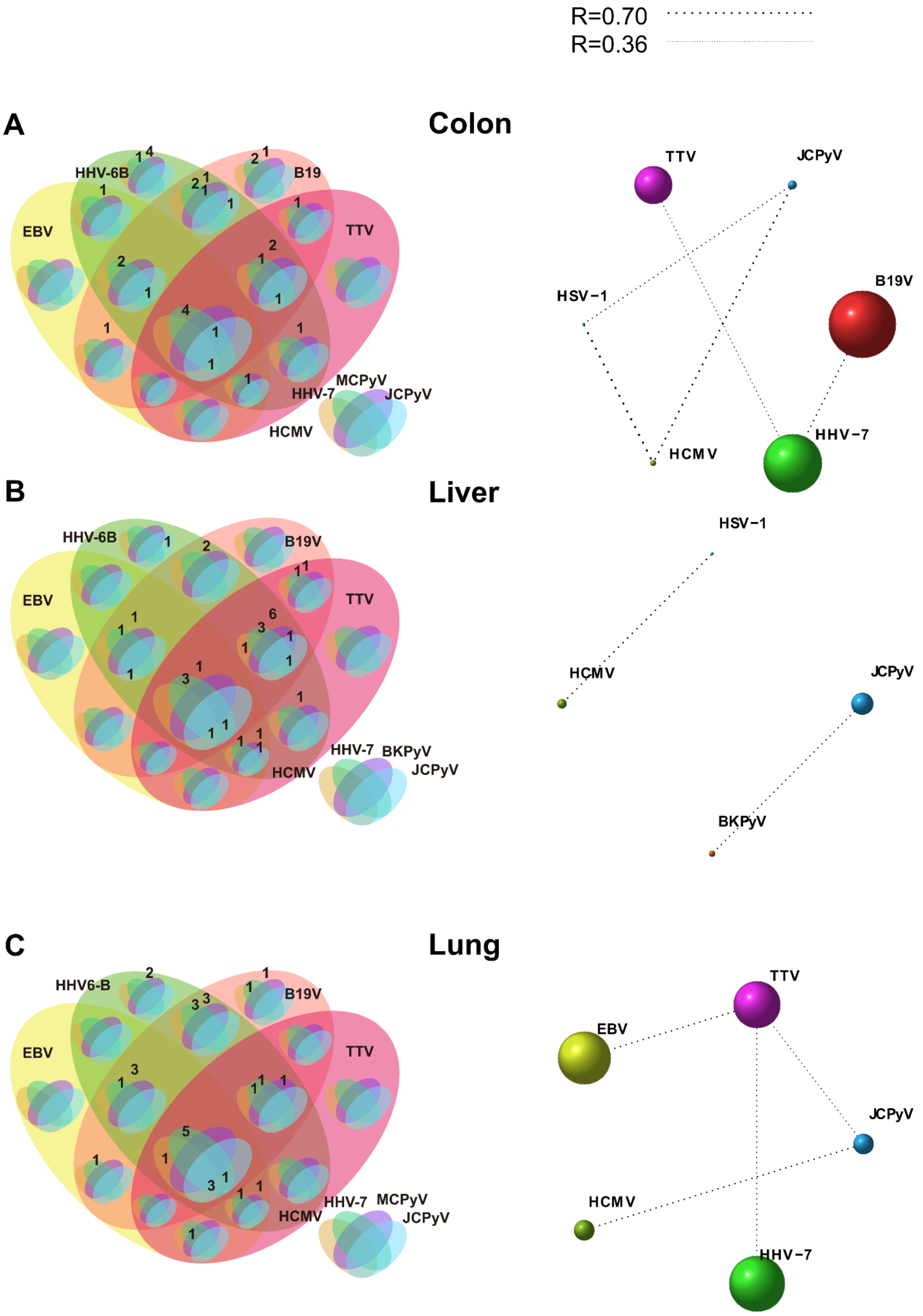

R=0.70
R=0.36
A
Colon
HHV-6B
B19
EBV
TTV
MCPyV
HHV-7
JCPyV
HCMV
TTV
JCPyV
HSV−1
B19V
HHV−7
HCMV
B
Liver
HHV-6B
B19V
EBV
TTV
HHV-7
BKPyV
JCPyV
HCMV
HSV−1
HCMV
JCPyV
BKPyV
C
Lung
HHV6-B
B19V
EBV
TTV
HHV-7
MCPyV
JCPyV
HCMV
TTV
EBV
JCPyV
HCMV
HHV−7

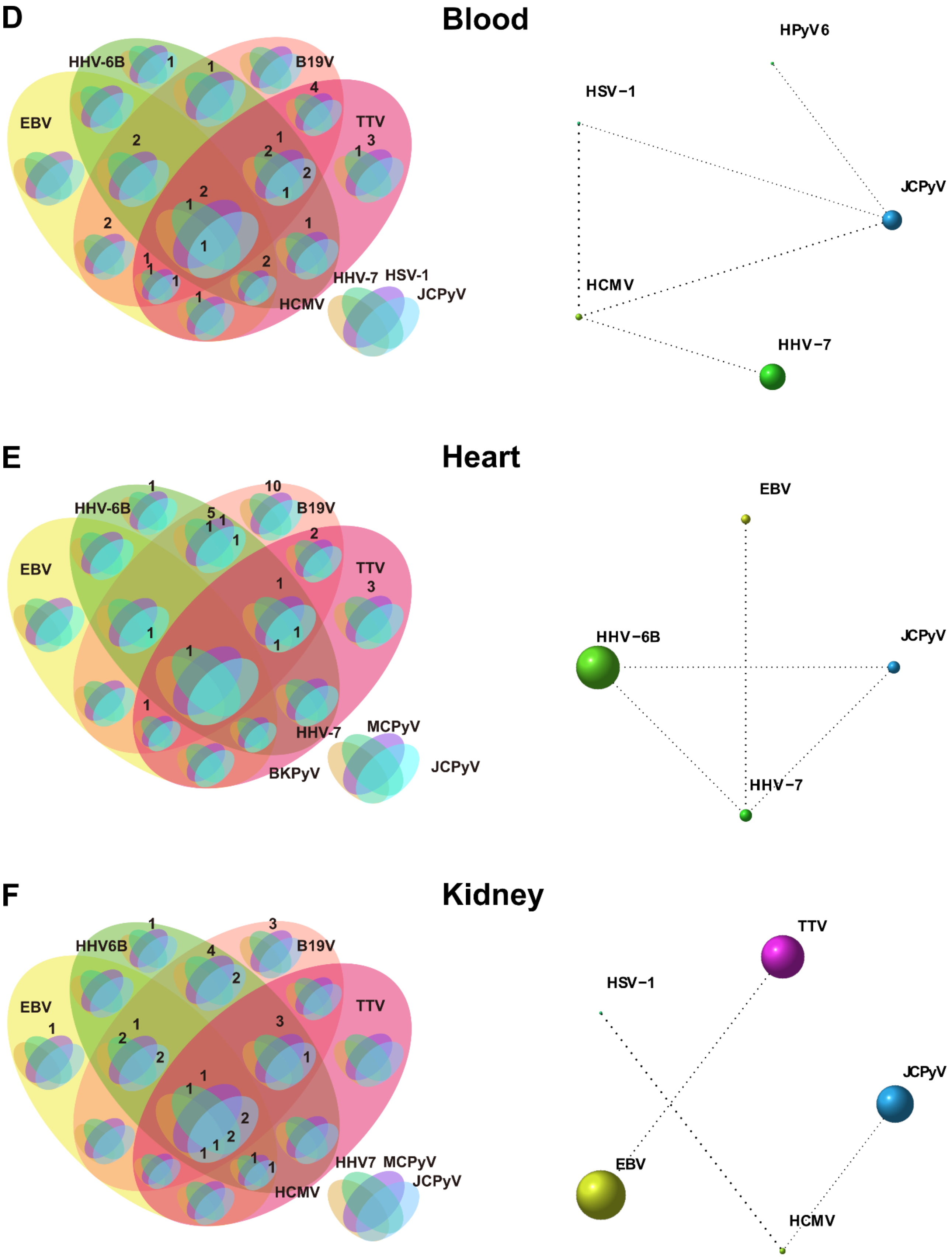
D
HHV-6B
B19V
EBV
TTV
HHV-7
HSV-1
JCPyV
HCMV
Blood
HPyV6
HSV−1
JCPyV
HCMV
HHV−7
E
HHV-6B
B19V
EBV
TTV
HHV-7
MCPyV
JCPyV
BKPyV
Heart
EBV
HHV−6B
JCPyV
HHV−7
F
HHV6B
B19V
EBV
TTV
HHV7
MCPyV
JCPyV
HCMV
Kidney
TTV
HSV−1
JCPyV
EBV
HCMV

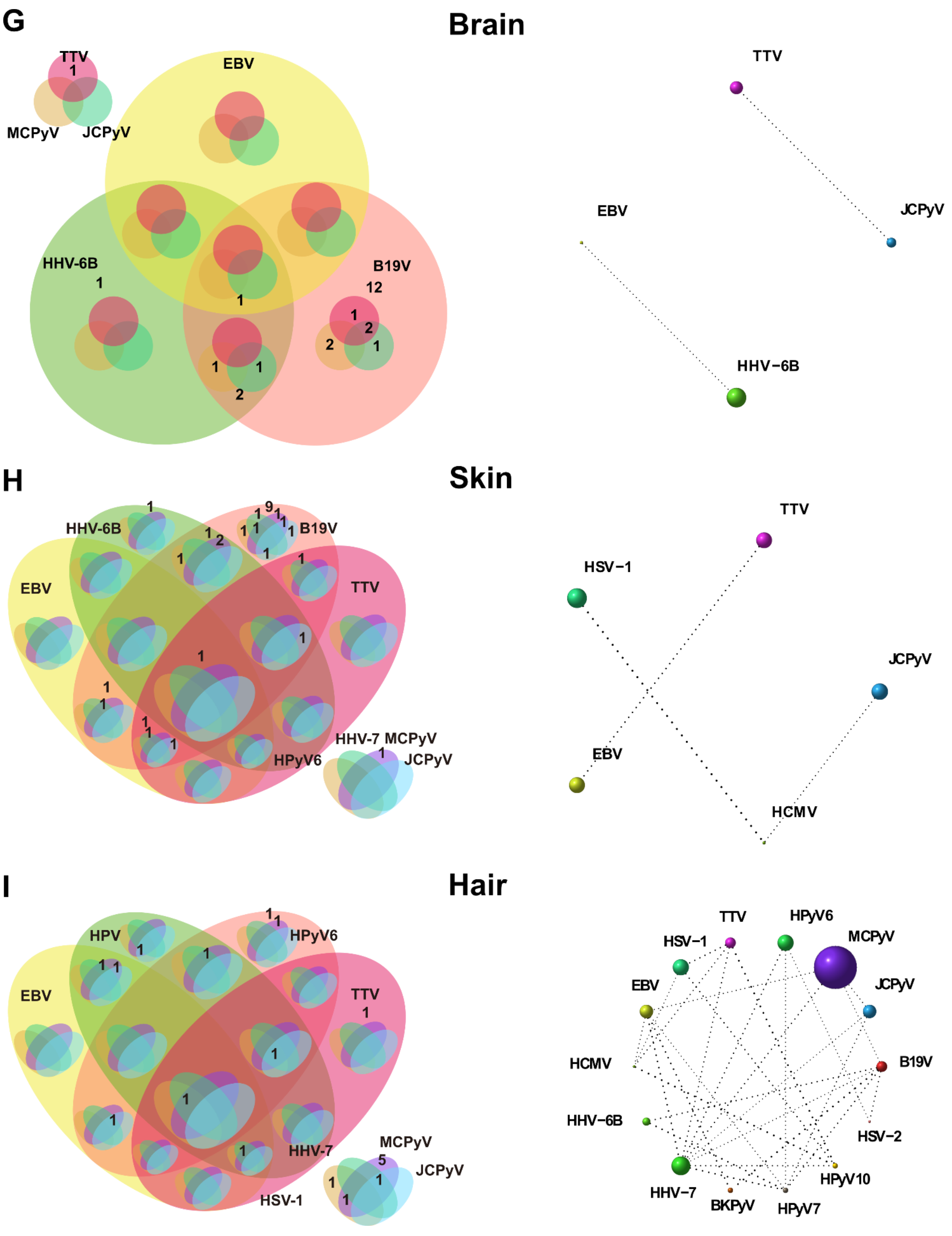

Supplementary Figure S5. Viral DNA co-occurrences in different organs. Venn diagrams on the left illustrate the number of co-occurring and isolated viral DNA findings in each tissue. On the right are the weighted correlation networks of co-existing viral DNAs calculated by φ coefficient ($p<0.05$). A dashed line between viruses represents that viral co-occurrence was statistically significant in that organ. Correlation strength is indicated by the width of the line. The sizes of the spheres represent viral DNA prevalence in a given organ.

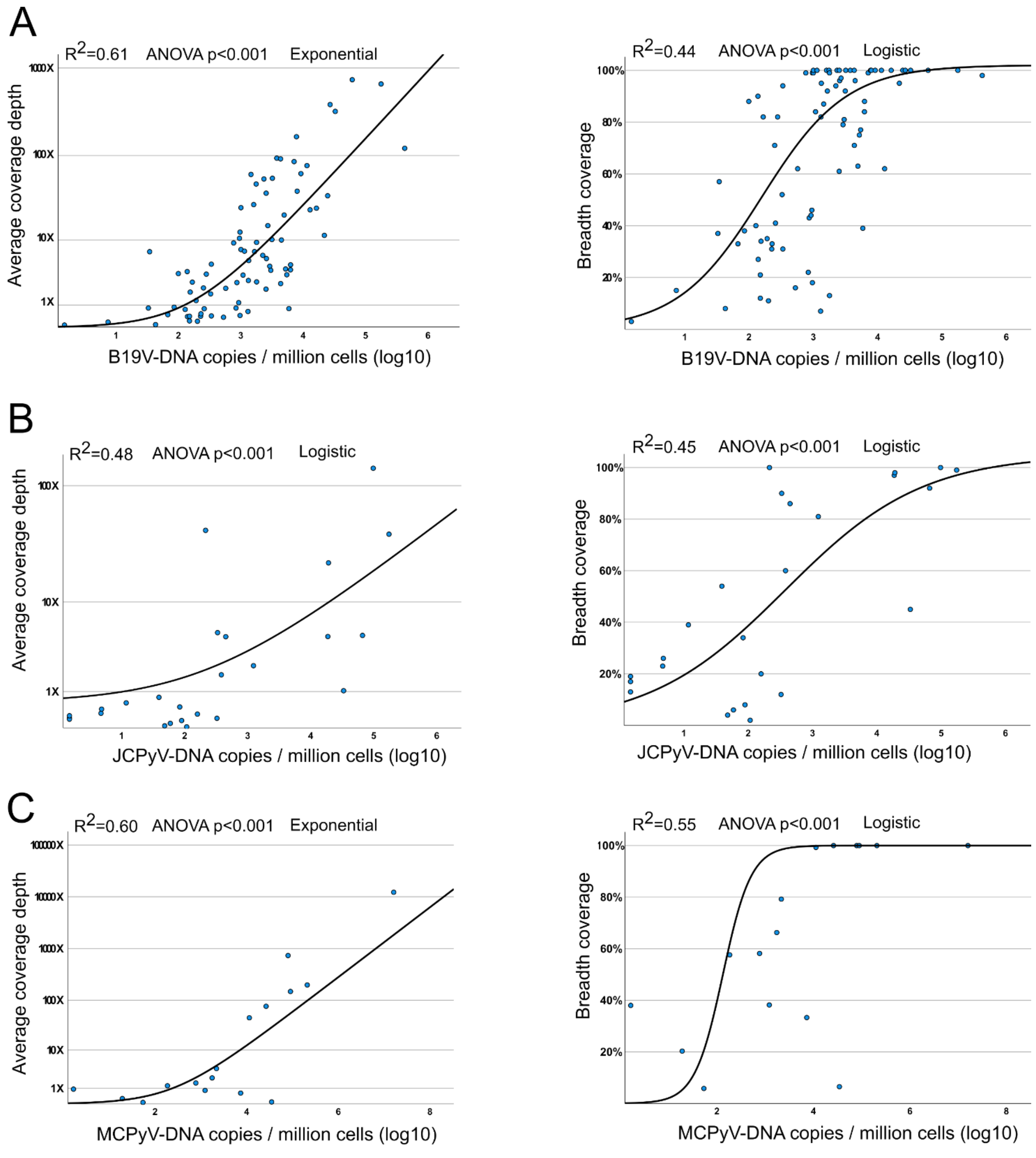
A
R^2=0.61 ANOVA p<0.001 Exponential
Average coverage depth
B19V-DNA copies / million cells (log10)
R^2=0.44 ANOVA p<0.001 Logistic
Breadth coverage
B19V-DNA copies / million cells (log10)
B
R^2=0.48 ANOVA p<0.001 Logistic
Average coverage depth
JCPyV-DNA copies / million cells (log10)
R^2=0.45 ANOVA p<0.001 Logistic
Breadth coverage
JCPyV-DNA copies / million cells (log10)
C
R^2=0.60 ANOVA p<0.001 Exponential
Average coverage depth
MCPyV-DNA copies / million cells (log10)
R^2=0.55 ANOVA p<0.001 Logistic
Breadth coverage
MCPyV-DNA copies / million cells (log10)

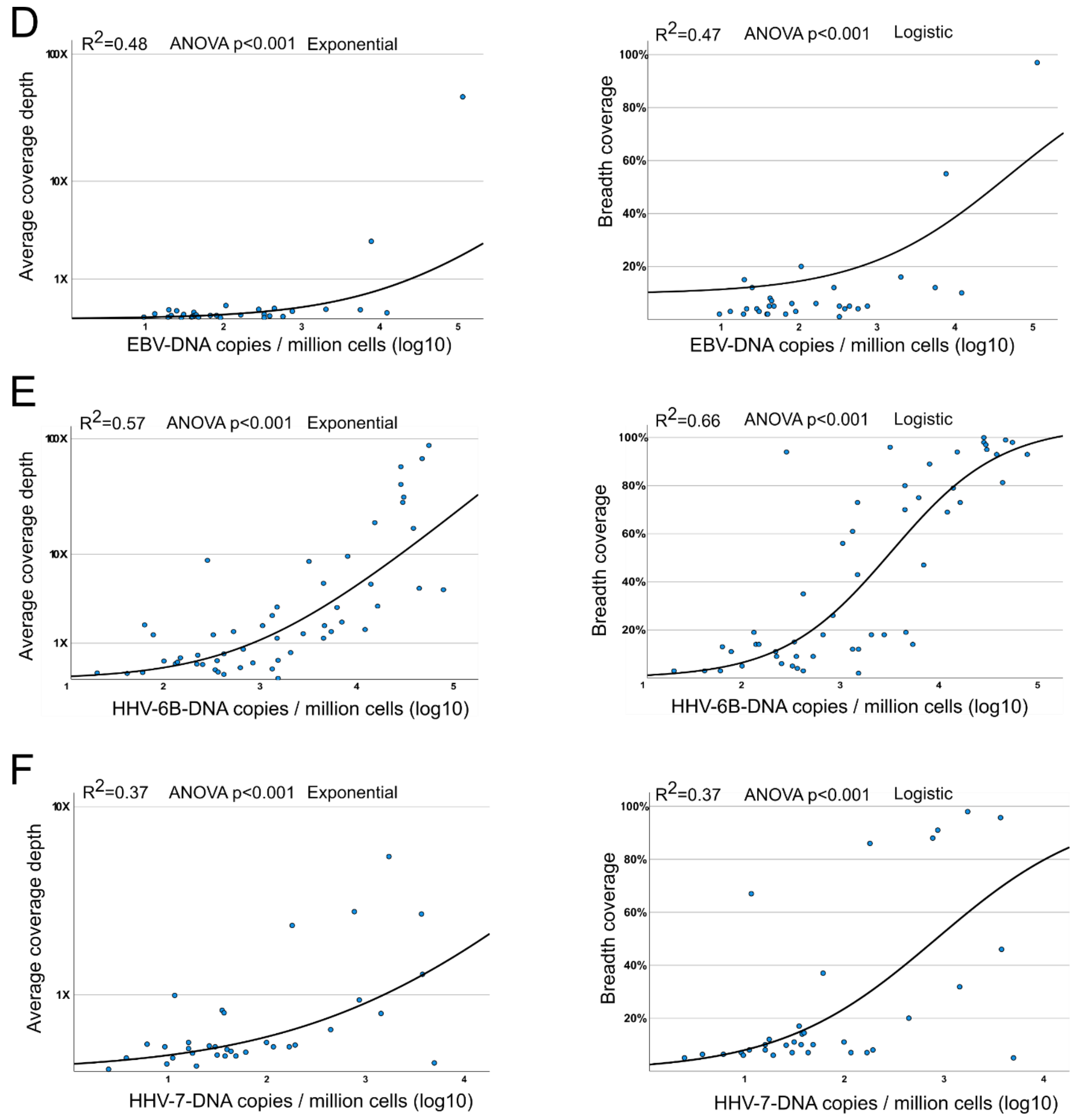

Supplementary Figure S6. Correlation of virus copies/million cells with mean coverage depth (left panel) and breadth (right panel). A) B19V, B) JCPyV, C) MCPyV D) EBV, E) HHV-6B, F) HHV-7. Results were statistically significant (p<0.001) with ANOVA. R^2 represents the coefficient of determination.

Supplementary Texts

Supplementary Text S1. Description of hybrid reference and de-novo-based genome reconstruction.

TRACESPipe pipeline uses alignment-based assembly together with *de novo* assembly to reconstruct genomes with maximum sensitivity and resolution (25). TRACESPipe selects the genome sequence with the highest breath coverage derived from five different rounds. The first round reconstructs a genome exclusively with an alignment-based approach according to the best reference. The second round uses the consensus generated from the alignments and aligns the *de novo* scaffolds using BWA (26) while prioritizing the reference-based approach. The third round is similar to round one, but priority is given to the *de novo* scaffolds. The alignments are produced with very high sensitivity, forcing the output to be more similar to the *de novo* when the consensus from the alignments is ambiguous or contains gaps. The fourth round finds the scaffolds from the *de novo* assembly with the highest similarities reported by FALCON-meta (27) and employs them as a candidate genome. The fifth round uses the scaffolds from round three as a reference and aligns the consensus sequence created in round one.

Supplementary Text S2. Computational controls to prevent irregular patterns, imbalanced representation, and exogenous content.

TRACESPipe includes three main controls: redundancy, database, and exogenous controls. These controls are critical to detect the source of irregular patterns, imbalanced representation, or exogenous content. The redundancy control estimates duplications or low-complexity regions in the sequences. TRACESPipe enables cross-checking of the information with similar sub-regions. GTO (28) is used to identify low-complexity regions (29), and it includes a DNA compressor that estimates the content along each genome based on GeCo2 (30). This information is crossed with the coverage profiles generated with BEDTools (31) and the data from the exogenous control. Finally, TRACESPipe removes duplicates using the markdup function from Samtools (32).

The database control includes viruses that share high similarity to other family members (e.g., Polyomaviridae) or the human host (e.g., Herpesviridae). These can result in imbalanced mapping of the reads to various references. When the references are complete genomes, the mapping automatically finds the best reference; however, when partial genomes are also included, the best reference may be attributed to a partial genome in which only conserved regions are present. To mitigate this, we apply FALCON-meta to measure the cross-similarity between the best references. Regarding the cross-similarity to human DNA, a small number of reads may be assigned to a reference virus, albeit of human origin. We apply FALCON-meta to measure and localize regions of high similarity between the viruses and human reference genome. Additionally, low-breadth coverage sequences are always manually inspected and confirmed by BLAST.

Fungi, bacteria, plants, archaea, and protozoa (among others), may display low levels of similarity to the viral or mitogenomes (33). TRACESPipe estimates the content of exogenous sequences with FALCON-meta (34) using databases for each respective type. The download and construction of the reference databases are automatically created with Entrez (35). The most representative genomes are aligned according to the respective reference for further impact identification on the reconstructed genomes.

Supplementary reference list

1. Moustafa,A., Xie,C., Kirkness,E., Biggs,W., Wong,E., Turpaz,Y., Bloom,K., Delwart,E., Nelson,K.E., Venter,J.C., *et al.* (2017) The blood DNA virome in 8,000 humans. *PLoS Pathog.*, **13**, e1006292.

2. Young,J.C., Chehoud,C., Bittinger,K., Bailey,A., Diamond,J.M., Cantu,E., Haas,A.R., Abbas,A., Frye,L., Christie,J.D., *et al.* (2015) Viral metagenomics reveal blooms of anelloviruses in the respiratory tract of lung transplant recipients. *Am. J. Transplant.*, **15**, 200–209.

3. Abbas,A.A., Diamond,J.M., Chehoud,C., Chang,B., Kotzin,J.J., Young,J.C., Imai,I., Haas,A.R., Cantu,E., Lederer,D.J., *et al.* (2017) The Perioperative Lung Transplant Virome: Torque Teno Viruses Are Elevated in Donor Lungs and Show Divergent Dynamics in Primary Graft Dysfunction. *Am. J. Transplant.*, **17**, 1313–1324.

4. Foulongne,V., Sauvage,V., Hebert,C., Dereure,O., Cheval,J., Gouilh,M.A., Pariente,K., Segondy,M., Burguière,A., Manuguerra,J.C., *et al.* (2012) Human skin Microbiota: High diversity of DNA viruses identified on the human skin by high throughput sequencing. *PLoS One*, **7**, e38499.

5. Wylie,K.M., Mihindukulasuriya,K.A., Zhou,Y., Sodergren,E., Storch,G.A. and Weinstock,G.M. (2014) Metagenomic analysis of double-stranded DNA viruses in healthy adults. *BMC Med.*, **12**, 71.

6. Tirosh,O., Conlan,S., Deming,C., Lee-Lin,S.Q., Huang,X., Barnabas,B.B., Bouffard,G.G., Brooks,S.Y., Marfani,H., Dekhtyar,L., *et al.* (2018) Expanded skin virome in DOCK8-deficient patients. *Nat. Med.*, **24**, 1815–1821.

7. Hall,J.B., Cong,Z., Imamura-Kawasawa,Y., Kidd,B.A., Dudley,J.T., Thiboutot,D.M. and Nelson,A.M. (2018) Isolation and Identification of the Follicular Microbiome: Implications for Acne Research. *J. Invest. Dermatol.*, **138**, 2033–2040.

8. Finkbeiner,S.R., Allred,A.F., Tarr,P.I., Klein,E.J., Kirkwood,C.D. and Wang,D. (2008) Metagenomic analysis of human diarrhea: Viral detection and discovery. *PLoS Pathog.*, **4**, e1000011.

9. Phan,T.G., Vo,N.P., Bonkoungou,I.J.O., Kapoor,A., Barro,N., O'Ryan,M., Kapusinszky,B., Wang,C. and Delwart,E. (2012) Acute Diarrhea in West African Children: Diverse Enteric Viruses and a Novel Parvovirus Genus. *J. Virol.*, **86**, 11024–11030.

10. Smits,S.L., Schapendonk,C.M.E., van Beek,J., Vennema,H., Schürch,A.C., Schipper,D., Bodewes,R., Haagmans,B.L., Osterhaus,A.D.M.E. and Koopmans,M.P. (2014) New viruses in idiopathic human diarrhea cases, the Netherlands. *Emerg. Infect. Dis.*, **20**, 1218–1222.

11. Holtz,L.R., Cao,S., Zhao,G., Bauer,I.K., Denno,D.M., Klein,E.J., Antonio,M., Stine,O.C., Snelling,T.L., Kirkwood,C.D., *et al.* (2014) Geographic variation in the eukaryotic virome of human diarrhea. *Virology*, **468**, 556–564.

12. Yinda,C.K., Vanhulle,E., Conceição-Neto,N., Beller,L., Deboutte,W., Shi,C., Ghogomu,S.M., Maes,P., Van Ranst,M. and Matthijnssens,J. (2019) Gut Virome Analysis of Cameroonians Reveals High Diversity of Enteric Viruses, Including Potential Interspecies Transmitted Viruses. *mSphere*, **4**, e00585-18.

13. Santiago-Rodriguez,T.M., Ly,M., Bonilla,N. and Pride,D.T. (2015) The human urine virome in association with urinary tract infections. *Front. Microbiol.*, **6**, 14.

14. Rani,A., Ranjan,R., McGee,H.S., Metwally,A., Hajjiri,Z., Brennan,D.C., Finn,P.W. and Perkins,D.L. (2016) A diverse virome in kidney transplant patients contains multiple viral subtypes with distinct polymorphisms. *Sci. Rep.*, **6**, 33327.

15. Ghose,C., Ly,M., Schwanemann,L.K., Shin,J.H., Atab,K., Barr,J.J., Little,M., Schooley,R.T., Chopyk,J. and Pride,D.T. (2019) The Virome of Cerebrospinal Fluid: Viruses Where We Once Thought There Were None. *Front. Microbiol.*, **10**, 2061.

16. Toppinen,M., Norja,P., Aaltonen,L.M., Wessberg,S., Hedman,L., Söderlund-Venermo,M. and Hedman,K. (2015) A new quantitative PCR for human parvovirus B19 genotypes. *J. Virol. Methods*, **218**, 40–45.

17. Pyöriä,L., Jokinen,M., Toppinen,M., Salminen,H., Vuorinen,T., Hukkanen,V., Schmotz,C., Elbasani,E., Ojala,P.M., Hedman,K., *et al.* (2020) HERQ-9 Is a New Multiplex PCR for Differentiation and Quantification of All Nine Human Herpesviruses. *mSphere*, **5**, e00265-20.

18. Toppinen,M., Pratas,D., Väisänen,E., Söderlund-Venermo,M., Hedman,K., Perdomo,M.F. and Sajantila,A. (2020) The landscape of persistent human DNA viruses in femoral bone. *Forensic Sci. Int. Genet.*, **48**, 102353.

19. Hoffman,N.G., Cook,L., Atienza,E.E., Limaye,A.P. and Jerome,K.R. (2008) Marked variability of BK virus load measurement using quantitative real-time PCR among commonly used assays. *J. Clin. Microbiol.*, **46**, 2671–2680.

20. Dumoulin,A. and Hirsch,H.H. (2011) Reevaluating and optimizing polyomavirus BK and JC real-time PCR assays to detect rare sequence polymorphisms. *J. Clin. Microbiol.*, **49**, 1382–1388.

21. Goh,S., Lindau,C., Tiveljung-Lindell,A. and Allander,T. (2009) Merkel cell polyomavirus in respiratory tract secretions. *Emerg. Infect. Dis.*, **15**, 489–491.

22. Antonsson,A., Bialasiewicz,S., Rockett,R.J., Jacob,K., Bennett,I.C. and Sloots,T.P. (2012) Exploring the prevalence of ten polyomaviruses and two herpes viruses in breast cancer. *PLoS One*, **7**, 39842.

23. Siebrasse,E.A., Reyes,A., Lim,E.S., Zhao,G., Mkakosya,R.S., Manary,M.J., Gordon,J.I. and Wang,D. (2012)

Identification of MW Polyomavirus, a Novel Polyomavirus in Human Stool. *J. Virol.*, **86**, 10321–10326.

24. Sadeghi,M., Wang,Y., Ramqvist,T., Aaltonen,L.M., Pyöriä,L., Toppinen,M., Söderlund-Venermo,M. and Hedman,K. (2017) Multiplex detection in tonsillar tissue of all known human polyomaviruses. *BMC Infect. Dis.*, **17**, 1–8.
25. Pratas,D., Toppinen,M., Pyoria,L., Hedman,K., Sajantila,A. and Perdomo,M.F. (2020) A hybrid pipeline for reconstruction and analysis of viral genomes at multi-organ level. *Gigascience*, **9**, 1–11.
26. Li,H. and Durbin,R. (2009) Fast and accurate short read alignment with Burrows-Wheeler transform. *Bioinformatics*, **25**, 1754–1760.
27. Pratas,D. and Pinho,A.J. (2018) Metagenomic composition analysis of sedimentary ancient DNA from the Isle of Wight. *Eur. Signal Process. Conf.*, **2018**-**Septe**, 1177–1181.
28. Almeida,J.R., Pinho,A.J., Oliveira,J.L., Fajarda,O. and Pratas,D. (2020) GTO: A toolkit to unify pipelines in genomic and proteomic research. *SoftwareX*, **12**, 100535.
29. Pinho,A.J., Garcia,S.P., Pratas,D. and Ferreira,P.J.S.G. (2013) DNA sequences at a glance. *PLoS One*, **8**, e79922.
30. Pratas,D., Hosseini,M. and Pinho,A.J. (2020) GeCo2: An Optimized Tool for Lossless Compression and Analysis of DNA Sequences. *Adv. Intell. Syst. Comput.*, **1005**, 137–145.
31. Quinlan,A.R. (2014) BEDTools: The Swiss-Army tool for genome feature analysis. *Curr. Protoc. Bioinforma.*, **2014**, 11.12.1-11.12.34.
32. Li,H., Handsaker,B., Wysoker,A., Fennell,T., Ruan,J., Homer,N., Marth,G., Abecasis,G. and Durbin,R. (2009) The Sequence Alignment/Map format and SAMtools. *Bioinformatics*, **25**, 2078–2079.
33. Budowle,B., Connell,N.D., Bielecka-Oder,A., Colwell,R.R., Corbett,C.R., Fletcher,J., Forsman,M., Kadavy,D.R., Markotic,A., Morse,S.A., *et al.* (2014) Validation of high throughput sequencing and microbial forensics applications. *Investig. Genet.*, **5**, 1–18.
34. Pratas,D., Hosseini,M., Grilo,G., Pinho,A.J., Silva,R.M., Caetano,T., Carneiro,J. and Pereira,F. (2018) Metagenomic composition analysis of an ancient sequenced polar bear jawbone from Svalbard. *Genes (Basel).*, **9**, 445.
35. Wheeler,D.L., Barrett,T., Benson,D.A., Bryant,S.H., Canese,K., Chetvernin,V., Church,D.M., DiCuccio,M., Edgar,R., Federhen,S., *et al.* (2007) Database resources of the National Center for Biotechnology Information. *Nucleic Acids Res.*, **35**, D5–D12.
